# *Atoh1* drives the heterogeneity of the pontine nuclei neurons and promotes their differentiation

**DOI:** 10.1126/sciadv.adg1671

**Published:** 2023-06-30

**Authors:** Sih-Rong Wu, Jessica C. Butts, Matthew S. Caudill, Jean-Pierre Revelli, Ryan S. Dhindsa, Mark A. Durham, Huda Y. Zoghbi

**Affiliations:** ^1^ Department of Neuroscience, Baylor College of Medicine, Houston, TX, USA.; ^2^ Jan and Dan Duncan Neurological Research Institute at Texas Children’s Hospital, Houston, TX, USA.; ^3^ Department of Molecular and Human Genetics, Baylor College of Medicine, Houston, TX, USA.; ^4^ Howard Hughes Medical Institute, Baylor College of Medicine, Houston, TX, USA.; ^5^ Program in Developmental Biology, Baylor College of Medicine, Houston, TX, USA.; ^6^ Medical Student Scientist Training Program, Baylor College of Medicine, Houston, TX, USA.; ^7^ Department of Pediatrics, Baylor College of Medicine, Houston, TX, USA.

## Abstract

Pontine nuclei (PN) neurons mediate the communication between the cerebral cortex andthe cerebellum to refine skilled motor functions. Prior studies showed that PN neurons fall into two subtypes based on their anatomic location and region-specific connectivity, but the extent of their heterogeneity and its molecular drivers remain unknown. *Atoh1* encodes a transcription factor that is expressed in the PN precursors. We previously showed that partial loss of *Atoh1* function in mice results in delayed PN development and impaired motor learning. In this study, we performed single-cell RNA sequencing to elucidate the cell state–specific functions of *Atoh1* during PN development and found that *Atoh1* regulates cell cycle exit, differentiation, migration, and survival of PN neurons. Our data revealed six previously not known PN subtypes that are molecularly and spatially distinct. We found that the PN subtypes exhibit differential vulnerability to partial loss of *Atoh1* function, providing insights into the prominence of PN phenotypes in patients with *ATOH1* missense mutations.

## Teaser

ScRNA-seq reveals that the Atoh1-dependent pontine nuclei, a hub for skilled motor function, are made up of six distinct subtypes.

## INTRODUCTION

Motor skills such as picking up a cup of coffee or hitting a speedy baseball with a bat require communication between the cerebral cortex and the cerebellum (*1*). These connections are mediated through the pontine nuclei (PN) that are located in the ventral pons and composed of mostly glutamatergic neurons (*2*–*7*). Given their pivotal role in motor functions, several efforts have been made to understand how the PN develop in mammals. PN neurons originate from a group of proliferating neuroepithelial cells residing in the rhombic lip (RL) located in the developing hindbrain. Specifically, *Wnt1-* and *Atoh1*-expressing cells within the caudal RL (cRL) give rise to glutamatergic PN neurons (*8*–*10*). PN neurons are born during mouse embryonic day 12.5 (E12.5) to E18.5 and migrate tangentially along the anterior extramural stream (AES) until they reach the ventral pons (*11*, *12*).

A previous study of PN in rabbit and cat has suggested that PN can be categorized into subpopulations on the basis of their anatomical location and connectivity (*13*). Anatomically, the PN are divided into the basal pontine nucleus (BPN) and the reticulotegmental nucleus (RtTg) at the anterior-ventral and posterior-dorsal part of the PN, respectively. Subpopulations of PN neurons have been proposed on the basis of their positions along the rostro-caudal axis, which inherit the expression pattern of *Hox2-Hox5* from their progenitors at the cRL (*14*). Moreover, tracing experiments demonstrated that the corticopontine connectivity was established in a partially region-specific manner (*15*, *16*), suggesting that PN neurons are functionally diverse on the basis of their regional cortical inputs. However, the extent of PN heterogeneity remains unclear, and the molecular determinants of PN heterogeneity are not known.

Another outstanding question is how a pool of seemingly homogenous *Atoh1*^+^ progenitors give rise to diverse progenies in the PN. *Atoh1*, or *Atonal homolog 1*, encodes a basic helix-loop-helix transcription factor ATOH1 that is required for the development of a variety of neurons in the hindbrain (*9*) and the dorsal spinal cord (*17*), hair cells in the inner ear (*18*), Merkel cells in the skin (*19*), and secretory cells in the gut (*20*). In the hindbrain, *Atoh1*-lineage neurons contribute to many key components of the proprioceptive pathway, including the PN (*9*, *21*). A recent report implicated a homozygous missense variant in *ATOH1* in two human patients with global developmental delay, motor function deficits, pontocerebellar hypoplasia, and hearing loss (*22*). It is thus of great interest and clinical relevance to understand how *Atoh1* shapes proper PN development.

Loss of both copies of *Atoh1* in mice results in complete absence of the PN, whereas loss of one copy of *Atoh1* in mice has no observable change in the PN (*23*), making it difficult to study the function of *Atoh1* during PN development using either the *Atoh1* knockout or the heterozygous mouse model. We previously found that substituting the serine at position 193 with an alanine (S193A) resulted in an *Atoh1* hypomorphic allele (*24*). Mice carrying *Atoh1^S193A^* over an *Atoh1* null allele (*Atoh1^S193A/−^*) showed delayed development in the PN neurons at postnatal day 0 (P0) and impaired motor learning as adults. Given the heterogeneity of the PN neurons, it is unclear whether this hypomorphic mutation affects the development of all PN subtypes equally. In other contexts, we learn that different cell types have differential vulnerability to perturbation in genes implicated in neurodevelopmental disorders (*25*, *26*). For example, despite being expressed in both neurogenic niches, increased level of the transcription factor *Foxg1* compromises the neurogenesis of excitatory neurons but not inhibitory neurons (*26*). We therefore hypothesized that PN subtypes are molecularly distinct and have differential vulnerability to partial loss of function of *Atoh1*.

In this study, we profiled the single-cell transcriptomes of the PN neurons and characterized the molecular and cellular phenotypes of the PN in *Atoh1^S193A/−^* mice at the single-cell resolution. We found cell state–specific roles of *Atoh1* during PN development including regulating cell cycle exit, differentiation, migration, survival, and cellular heterogeneity of the PN neurons. In addition, our single-cell RNA sequencing (scRNA-seq) data revealed six unreported subtypes of the PN neurons at P5 and identified subtype-specific markers. We found that the PN subtypes have differential vulnerabilities to partial loss of function of *Atoh1*. Our study provides comprehensive evidence of how a transcription factor plays multiple roles during neuronal development and demonstrates that neuronal subtypes could have differential sensitivity to perturbation of a gene that is expressed in the precursors of all subtypes.

## RESULTS

### Phospho-mutation of *Atoh1* at serine-193 leads to PN hypoplasia in mice

**Fig. 1.**
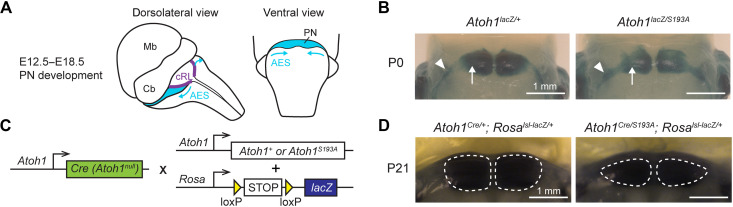
Phospho-mutation of *Atoh1* at serine-193 leads to PN hypoplasia in mice. (**A**) Schematic representation of the PN development in mice. PN neurons migrate from cRL to ventral pons through AES during E12.5 to E18.5. Mb, midbrain; Cb, cerebellum. (**B**) Whole-mount X-galactosidase (X-gal) staining on mouse hindbrain at P0 (ventral view). The arrows and arrowheads denote the neurons at the PN and in the AES, respectively. Scale bars, 1 mm. (**C**) Strategy to label the *Atoh1*-lineage neurons. *Atoh1^Cre/+^* knock-in mouse was crossed with mouse carrying either wild-type or hypomorphic *Atoh1* (*Atoh1^S193A^*) and a Cre-dependent *lacZ* allele. (**D**) Whole-mount X-gal staining on mouse hindbrain at P21 (ventral view). The dashed lines outline the area of the PN. Scale bars, 1 mm.

To test whether partial loss of function of *Atoh1* results in PN abnormalities postnatally, we compared the morphology of the PN in mice with hypomorphic mutation to those in control mice at P0 and P21. We crossed *Atoh1^S193A/+^* mice to *Atoh1^lacZ/+^*, whereby the *lacZ* allele replaced the coding region of *Atoh1*, creating a null allele. Following the tangential migration along the AES (Fig. 1A), most of the PN neurons finished migrating at P0 in control mice (Fig. 1B, left, arrow), with little lacZ signal in the AES (arrowhead). We observed a decreased intensity of the lacZ staining in the PN in *Atoh1^S193A/lacZ^* mice (Fig. 1B, right, arrow), indicating potential neuronal loss at P0 in *Atoh1^S193A/lacZ^* mice. In addition, consistent with our previous study (*24*), we found that there were more *lacZ*^+^ neurons retained at the AES in *Atoh1^S193A/lacZ^* mice (Fig. 1B, right, arrowhead), suggesting a developmental delay or a migration deficit in some PN neurons upon partial loss of *Atoh1* function.

To test whether those neurons retained at the AES ever reach their destination, we examined the PN morphology at a juvenile age, P21. Given that *Atoh1* is turned off postnatally in the PN, we permanently labeled the *Atoh1*-lineage neurons using a Cre-dependent lacZ reporter (*27*) in combination with an *Atoh1^Cre/+^* knock-in mouse in which one copy of *Atoh1* is functionally a null allele because the Cre replaced the coding region of *Atoh1* (*28*). This allows us to visualize the PN postnatally (Fig. 1C). We found that the size of the PN was reduced in *Atoh1^Cre/S193A^; Rosa^lsl-lacZ/+^* animals compared to *Atoh1^Cre/+^; Rosa^lsl-lacZ/+^* animals (Fig. 1D). We further confirmed this phenotype quantitatively by performing immunofluorescence staining with osteopontin antibody, a pan-PN neuronal marker (fig. S1). These data suggested that partial loss of function of *Atoh1* not only leads to delayed development of PN but also leads to PN hypoplasia in mice reminiscent of the malformation of the PN in patients with *ATOH1* missense variant (*22*). Together, these data reinforced the importance of *Atoh1* in PN development and demonstrated that *Atoh1* hypomorphic allele could provide a useful mouse model to dissect the functions of *Atoh1* during PN development.

### Progression of the PN development is impaired in *Atoh1^S193A/−^* animals

**Fig. 2.**
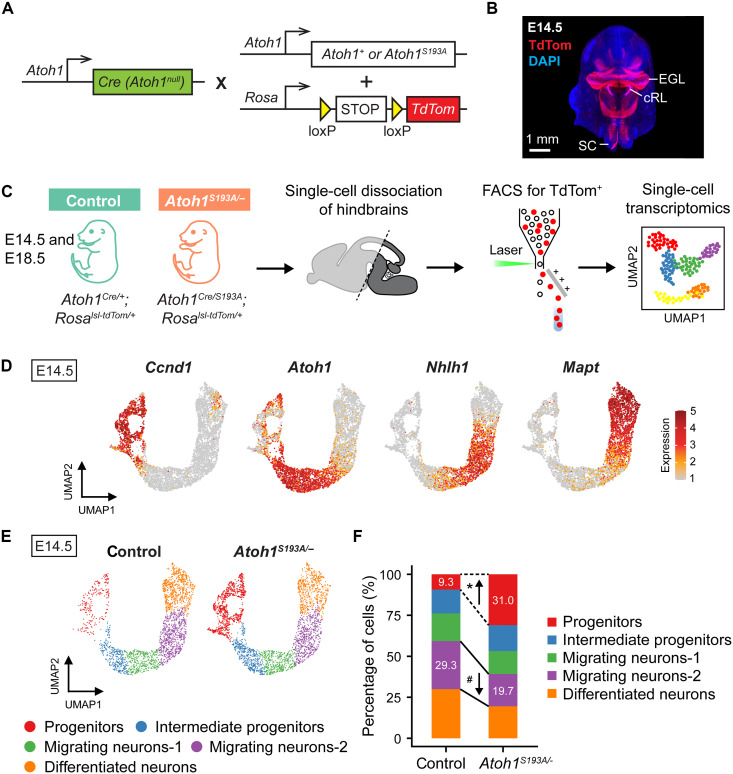
*Atoh1^S193A/−^* animals exhibited an impaired progression of PN development. (**A**) Strategy to label the *Atoh1*-lineage neurons. *Atoh1^Cre/+^* knock-in mouse was crossed with mouse carrying either wild-type or hypomorphic *Atoh1* (*Atoh1^S193A^*) and a Cre-dependent *TdTomato* (*TdTom*) reporter. (**B**) An image of three-dimensional rendering of E14.5 mouse head using lightsheet microscopy (back view). The TdTom represents the *Atoh1*-lineage neurons in developing hindbrain. EGL, external granule layer; SC, spinal cord. (**C**) Workflow for scRNA-seq of *Atoh1*-lineage neurons from E14.5 and E18.5 mouse hindbrain. Hindbrains were collected from E14.5 and E18.5 control and *Atoh1^S193A/−^* embryos (*n* = 3 per genotype for each time point). After enrichment by sorting, single-cell transcription profiles were captured by 10X Genomics Chromium platform. (**D**) Expression levels of the selective markers visualized on uniform manifold approximation and projection (UMAP). *Ccnd1*, *Atoh1*, *Nhlh1*, and *Mapt* are known markers for progenitors, intermediate progenitors, migrating neurons, and differentiated neurons, respectively. (**E**) UMAP of the five cell states identified in developing PN at E14.5 by scRNA-seq. (**F**) Proportion of the cells in each cell state. The arrows indicate the direction of the change in *Atoh1^S193A/−^* animals. *False discovery rate (FDR) < 0.01 and #FDR < 0.05.

To investigate the mechanisms by which *Atoh1* regulates normal PN development, we performed scRNA-seq in developing hindbrains from control mice and mice with *Atoh1* hypomorphic mutation. We used a Cre-dependent fluorescent reporter (*Rosa^lsl-TdTomato^*) to permanently label *Atoh1*-lineage neurons with *Atoh1^Cre/+^* mice (Fig. 2, A and B). During development, PN progenitors and migrating PN neurons are spatially dispersed between the cRL to ventral pons. Therefore, to ensure that we capture all PN progenitors and migrating neurons, we collected whole hindbrains from *Atoh1^Cre/+^; Rosa^lsl-tdTomato/+^* (hereafter referred to as control) and *Atoh1^Cre/S193A^; Rosa^lsl-tdTomato/+^* (hereafter referred to as *Atoh1^S193A/−^*) embryos. After single-cell dissociation, *Atoh1*-lineage (TdTom^+^) neurons were isolated by fluorescence- activated cell sorting (FACS), followed by scRNA-seq with 10X Genomics Chromium platform (Fig. 2C). We collected samples at two time points, E14.5 and E18.5, to examine the phenotypes of *Atoh1^S193A/−^* mice in the middle and at the end of neurogenesis of the PN neurons, respectively.

We first focused on the E14.5 time point to explore the role of *Atoh1* during PN development. After filtering out low-quality cells (see Materials and Methods), a total of 22,191 cells were retained from control hindbrains, and 22,206 cells were retained from *Atoh1^S193A/−^* hindbrains (*n* = 3 animals per genotype). Among the cells in the hindbrain, we identified the PN cells based on the clusters that expressed the established markers of the AES and PN (fig. S2, A to D). This resulted in 2451 control PN cells and 3124 *Atoh1^S193A/−^* PN cells for downstream analyses. After unbiased clustering of the PN cells, we annotated each cluster by the expression of the known markers (Fig. 2D, fig. S2E, and data S1). Cells at different cell states during PN development were fully captured in our dataset, including proliferating progenitors (*Ccnd1*^+^), postmitotic intermediate progenitors (*Atoh1*^high^), migrating neurons (*Nhlh1*^+^), and differentiated neurons (*Mapt*^+^) in both control and *Atoh1^S193A/−^* samples (Fig. 2E). The migrating neurons constituted the largest population and were divided into two clusters. We therefore annotated the two migrating populations as migrating neurons-1 and migrating neurons-2 based on their maturity. The migrating neurons-1 are neurons starting to differentiate and migrate away from the cRL (*Atoh1*^low^*Nhlh1*^high^), whereas the migrating neurons-2 are neurons expressing not only high level of the marker for migration but also low level of the differentiated neuronal marker (*Nhlh1*^high^*Mapt* ^low^) (Fig. 2, D and E).

Next, we calculated the percentage of the cells in each cell state in control and *Atoh1^S193A/−^* animals and performed differential abundance test using propeller with speckle R package (*29*). We found that the proportion of the progenitors increased markedly from 9.3 to 31% with a significant decrease in the percentage of the migrating neurons-2 from 29.3 to 19.7% in *Atoh1^S193A/−^* animals (Fig. 2F and fig. S3A). The shift in the proportion of the cells in each state indicates that the progression of the PN development was impaired in *Atoh1^S193A/−^* mice. Trajectory analysis with Slingshot (*30*, *31*) showed that in control embryos, more cells progressed further in pseudo-time than in *Atoh1^S193A/−^* embryos (fig. S3, B and C). Collectively, these data demonstrate that partial loss of *Atoh1* function leads to accumulation of the PN progenitors at the expense of the differentiating PN neurons.

### Partial loss of function of *Atoh1* leads to decreased cell cycle exit in PN progenitors and deficits in differentiation and migration

**Fig. 3.**
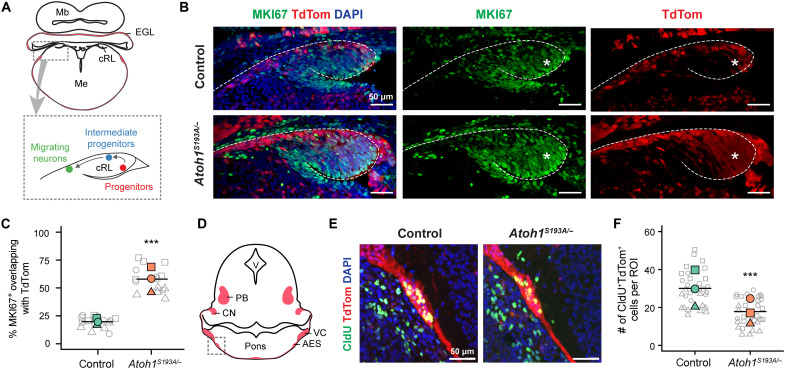
Partial loss of function of *Atoh1* leads to decreased cell cycle exit in PN progenitors and deficits in differentiation and migration. (**A**) Schematic illustration of mouse coronal section at E14.5. The area of cRL was enlarged on the bottom with locations of three cell states being labeled. Me, medulla. (**B**) Immunofluorescence staining of MKI67 on E14.5 mouse brain. The cropped view of cRL in control (top) and *Atoh1^S193A/−^* (bottom) mice. *Atoh1*-lineage neurons were labeled with TdTom, and the nuclei were labeled with 4′,6-diamidino-2-phenylindole (DAPI). The asterisk denotes the progenitors at cRL. Scale bars, 50 μm. (**C**) Quantification of the percentage of MKI67 fluorescence overlapping with TdTom in control and* Atoh1^S193A/−^* mice. The gray symbols represent the five regions of interest quantified from each animal (*n* = 3 per genotype). The average percentage for each animal was shown in colored shape. The crossbar denotes the mean per genotype. ***Χ^2^(1) = 53.95, *P* = 2.06 × 10^−13^ by mixed-model analysis of variance (ANOVA). (**D**) Schematic illustration of coronal section at E14.5. The dashed gray box indicates the region of interest shown in (E). V, ventricle; PB, parabrachial nuclei; CN, cerebellar nuclei; VC, ventral cochlear nucleus. (**E**) Immunofluorescence staining of CldU on E14.5 mouse brain. The areas of AES for control (left) and *Atoh1^S193A/−^* (right) animals were shown. *Atoh1*-lineage neurons were labeled with TdTom, and the nuclei were labeled with DAPI. Scale bars, 50 μm. (**F**) The average number of the CldU^+^TdTom^+^ cells per region of interest (ROI) in control and *Atoh1^S193A/−^* mice. The gray symbols represent the 10 regions of interest quantified from each animal (*n* = 3 per genotype). The average number for each animal was shown in colored shape. The crossbar denotes the mean per genotype. ***Χ^2^(1) = 39.81, *P* = 2.80 × 10^−10^ by mixed-model ANOVA.

We sought to investigate the mechanisms underlying the altered proportions of the cells in each state in *Atoh1^S193A/−^* animals. While we observed an increased proportion of the proliferating progenitors in *Atoh1^S193A/−^* mice, the proportion of its downstream cell state (i.e., the intermediate progenitors) was not altered (Fig. 2F). Instead, the fraction of the migrating neurons-2 was significantly decreased. Therefore, we proposed two mechanisms underlying the observed phenotypes: Robust function of *Atoh1* is required to (i) drive cell cycle exit and (ii) promote differentiation and migration (fig. S3D). To validate our scRNA-seq data, we performed immunofluorescence staining and histological analyses on the embryonic tissues. First, we performed immunofluorescence staining for MKI67, a marker for proliferating cells, on E14.5 hindbrains of control and *Atoh1^S193A/−^* animals. On the basis of the staining of ATOH1 and MKI67 at the cRL in control animals (fig. S4), the MKI67^+^ proliferating progenitors and ATOH1^+^ intermediate progenitors reside at the ventromedial and dorsolateral cRL, respectively (Fig. 3A). In control animals, there were only few TdTom^+^ cells at the cRL (Fig. 3B, asterisks), suggesting that the proliferating PN progenitors become postmitotic and leave the cRL upon the onset of *Atoh1* expression. In contrast, there was an increased percentage of the MKI67^+^ cells that overlapped with TdTom at the cRL in *Atoh1^S193A/−^* mice (Fig. 3, B and C), indicating an accumulation of proliferating progenitors at the cRL. The data suggest that robust *Atoh1* function is important for the cycling progenitors to become postmitotic.

Next, to test whether the differentiating and migrating neurons are reduced in *Atoh1^S193A/−^* mice, we used the thymidine analog, 5-chloro-2′-deoxyuridine (CldU) to label and quantify the number of PN neurons born within the 24-hour window between E13.5 and E14.5. We injected the pregnant dams with CldU at E13.5 and harvested the brains from E14.5 embryos. It has been shown that it takes 1 to 2 days for the PN neurons to migrate from cRL to ventral pons (*12*). Consistent with the literature, we found that most of the CldU-labeled PN neurons (CldU^+^TdTom^+^) were located at the AES 24 hours after injection in control animals (Fig. 3, D and E). The number of the CldU^+^TdTom^+^ cells at the AES was significantly reduced in *Atoh1^S193A/−^* mice (Fig. 3F), suggesting that fewer neurons differentiated and migrated away from the cRL in *Atoh1^S193A/−^* animals. These data validate our scRNA-seq data in which we found that migrating population was decreased in *Atoh1^S193A/−^* mice. Together, our data demonstrate that *Atoh1* governs multiple biological processes in addition to specifying PN neurons. *Atoh1* is important for both cell cycle exit of the PN progenitors and the differentiation and migration of the intermediate progenitors.

### The cell state–specific dysregulated pathways in *Atoh1^S193A/−^* mice

**Fig. 4.**
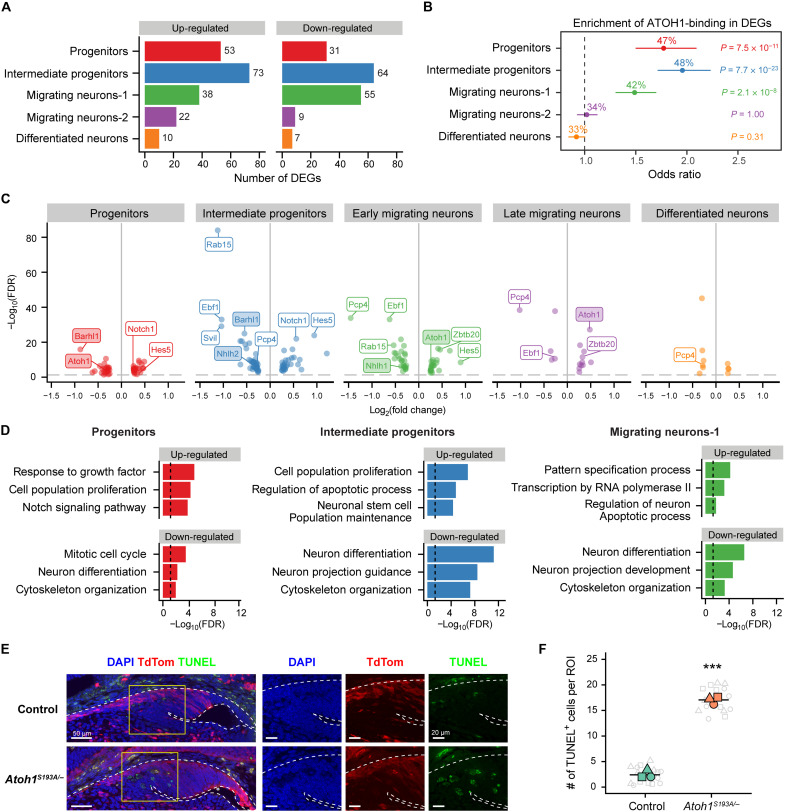
Partial loss of *Atoh1* function leads to multiple dysregulated pathways. (**A**) The number of the up-regulated (left) and down-regulated (right) genes at each cell state in *Atoh1^S193A/−^* compared to control. The DEGs were filtered by log_2_FC > 0.25 and FDR < 0.05. (**B**) The enrichment of the DEGs with ATOH1-binding in each cell state. The percentage of the DEGs with ATOH1-binding peak was shown in number. The odds ratios were presented with 95% confidence interval performed by Fisher’s exact test. *P* value was adjusted by Bonferroni. (**C**) Volcano plot of the DEGs with ATOH1-binding peak grouped by cell state. The shaded box indicates the known *Atoh1* targets that have been reported in PN development. (**D**) Gene ontology (GO) enrichment analysis of the DEGs. The representative biological processes are shown with −log_10_(FDR). (**E**) TUNEL staining on cRL in control (top) and *Atoh1^S193A/−^* (bottom) at E14.5. The *Atoh1*-lineage cells were labeled with TdTom, and the nuclei were stained with DAPI. (**F**) The average number of the TUNEL^+^ cells per region of interest in control and *Atoh1^S193A/−^* mice. The gray symbols represent the five ROIs quantified from each animal (*n* = 3 per genotype). The average number for each animal was shown in colored shape. The crossbar denotes the mean per genotype. ***Χ^2^(1) = 82.41, *P* = 2.20 × 10^−16^ by mixed-model ANOVA.

To understand how *Atoh1* mediates different biological processes during PN development, we sought to identify the genes that were dysregulated in each cell state in *Atoh1^S193A/−^* mice compared to control mice. Using differential gene expression analysis, we identified 362 differentially expressed genes (DEGs) [log_2_ fold change (log_2_FC) > 0.25 and false discovery rate (FDR) < 0.05] (data S2). We found that intermediate progenitors had the highest number of DEGs, followed by migrating neurons-1 and progenitors (Fig. 4A). We verified that the different number of DEGs per cell state was not simply a result of differences in total cell number in each cell state (fig. S5A). The cell states with the greatest transcriptional dysregulation (i.e., progenitors, intermediate progenitors, and migrating neurons-1) also expressed high levels of Atoh1 (Figs. 4A and 2D), which led us to test whether these DEGs were directly regulated by *Atoh1*. We calculated the percentage of the DEGs that had ATOH1 binding peak(s) identified by ATOH1 chromatin immunoprecipitation sequencing (*32*) and performed enrichment analysis for each cell state. We found a significant enrichment of DEGs with ATOH1-binding in progenitors (47%), intermediate progenitors (48%), and migrating neurons-1 (42%) (Fig. 4B), highlighting the direct impact of *Atoh1* in these cell states. We found several genes that were direct targets of *Atoh1* and have been reported to play a role in PN development (Fig. 4C, shaded box). For instance, *Atoh1*, which is known to regulate itself (*33*), was down-regulated in the progenitors. *Barhl1*, important for migration and survival of the PN neurons (*34*), was down-regulated in both progenitors and intermediate progenitors upon partial loss of *Atoh1* function. Last, *Nhlh1*, essential for migration of the PN neurons (*35*), was decreased in the intermediate progenitors and the migrating neurons. Moreover, we also identified other DEGs such as *Pcp4* and *Rab15*, whose roles have not been characterized in PN development but have robust changes in expression levels in multiple cell states (Fig. 4C). *Pcp4* has been identified as an *Atoh1* target in cochlear hair cells (*36*). *Rab15* was also reported as an *Atoh1* target in different *Atoh1*-lineage cells including developing cerebellar granule neurons (*32*), Merkel cells (*37*), cochlear hair cells (*36*), and neurons in dorsal neural tube (*38*).

Next, we focused on the three most affected cell states and performed gene ontology (GO) enrichment analysis to identify the pathways that were dysregulated in *Atoh1^S193A/−^* mice beyond the known *Atoh1* targets (Fig. 4D and data S3). In line with the finding of increased proliferating cells in *Atoh1^S193A/−^* mice, cell proliferation and cell cycle regulation including Notch signaling were among the top enriched GO terms for the up-regulated genes in the progenitor state (Fig. 4D, left). Consistent with the trajectory analysis that showed an impaired differentiation process in *Atoh1^S193A/−^* mice, down-regulated genes were enriched for neuron differentiation and cytoskeleton organization ontologies across all three cell states (Fig. 4D). These data supported our earlier findings that *Atoh1* plays roles in both cell cycle regulation, neuronal differentiation, and migration. We found that up-regulated genes in both intermediate progenitors and migrating neurons-1 were enriched for regulators of apoptosis (Fig. 4D, middle and right). For instance, the average level of *Hrk*, a member of proapoptotic *Bcl-2* family, was significantly increased in both intermediate progenitors and migrating neurons-1 in *Atoh1^S193A/−^* mice (fig. S5B). In addition, other genes involved in programmed cell death such as *Pak3* and *Dap* were also up-regulated in the intermediate progenitor state (fig. S5, C and D). These data indicate that *Atoh1* hypomorphic mutation might lead to increased cell death. To test this hypothesis, we performed terminal deoxynucleotidyl transferase–mediated deoxyuridine triphosphate nick end labeling (TUNEL) assay and found that the number of TUNEL-positive cells was significantly increased in *Atoh1^S193A/−^* mice at the lateral cRL (Fig. 4, E and F), where the intermediate progenitors (*Atoh1*^high^) are located (Fig. 3A and fig. S4). The increased cell apoptosis in *Atoh1^S193A/−^* animals may count for the reduced size of PN at older age and suggest that *Atoh1* is important for the survival of the intermediate progenitors. In summary, these data highlight the important roles of *Atoh1* in progenitors, intermediate progenitors, and migrating neurons by regulating cell cycle, differentiation, migration, and survival of the developing PN neurons.

### PN neurons are molecularly heterogeneous

**Fig. 5.**
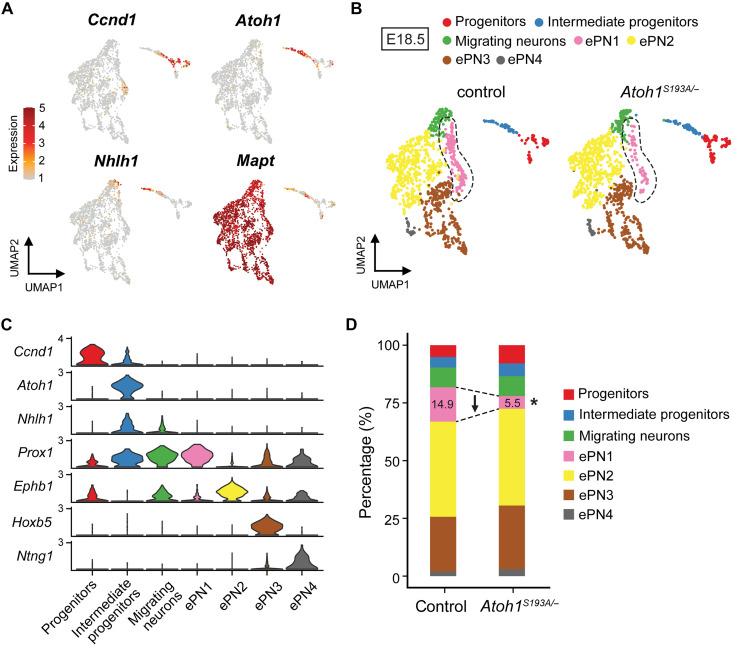
PN neurons at E18.5 are heterogeneous and exhibit differential vulnerability to *Atoh1* hypomorphic mutation. (**A**) Expression levels of the selective markers visualized on UMAP. *Ccnd1*, *Atoh1*, *Nhlh1*, and *Mapt* are known markers for progenitors, intermediate progenitors, migrating neurons, and differentiated neurons, respectively. (**B**) UMAP of the major cell states and ePN subtypes at E18.5. The dashed line denotes the subtype that was significantly reduced in *Atoh1^S193A/−^* mice. (**C**) Violin plot showing the expression levels of the marker genes. *Prox1*, *Ephb1*, *Hoxb5*, and *Ntng1* are the marker genes for ePN1, ePN2, ePN3, and ePN4, respectively. (**D**) Proportion of the cells in each cell state and PN subtype. The arrow indicates the direction of the change in *Atoh1^S193A/−^* animals. *FDR < 0.01.

To determine whether the cellular identities of the differentiated PN neurons are altered in *Atoh1^S193A/−^* mice, we performed scRNA-seq in control and *Atoh1^S193A/−^* hindbrains at E18.5, when most of the PN neurons have been born and migrated to the ventral pons. We analyzed 1196 control and 1176 *Atoh1^S193A/−^* PN cells (*n* = 3 animals per genotype) (fig. S6, A to D). After unbiased clustering, we annotated the clusters based on the known marker genes (Fig. 5, A and B). As expected, most of the cells at E18.5 are differentiated neurons marked by *Mapt* expression (Fig. 5A). The differentiated neurons were classified into four subtypes (Fig. 5B), suggesting that they are molecularly heterogeneous. We characterized the marker genes for each subtype based on differential gene expression analysis and named the differentiated PN neuron subtypes as embryonic PN1(ePN1) to ePN4 (Fig. 5C, fig. S6E, and data S1). Similar to the E14.5 data, we found a slightly increased proportion of the progenitors in *Atoh1^S193A/−^* mice at E18.5 (Fig. 5D). In addition, the percentage of ePN1 cells was significantly reduced from 14.9 to 5.5% (Fig. 5D and fig. S6F), while there was no significant change in other ePN subtypes. These data indicate that ePN subtypes have differential vulnerability to the *Atoh1* hypomorphic mutation.

**Fig. 6.**
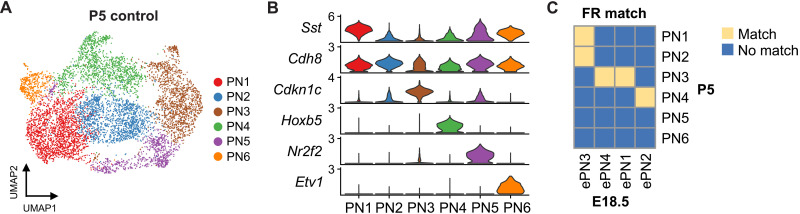
scRNA-seq reveals six PN subtypes in mice at P5. (**A**) UMAP of the major subtypes of the PN neurons at P5. (**B**) Violin plot showing the expression levels of the marker genes for each PN subtype. (**C**) Matching the cell type between E18.5 ePN subtypes and P5 PN subtypes by FR test.

To test whether the differential vulnerability of the PN subtypes in *Atoh1^S193A/−^* embryos proceeds to the postnatal stage, we first determined whether the PN neurons maintained their molecular heterogeneity at P5. We enriched the PN neurons by dissecting and pooling the PN from control mice at P5 (*n* = 11 to 13 per replicate) and performed scRNA-seq for TdTom^+^ cells. Six PN subtypes (PN1 to PN6) were uncovered using unbiased clustering (Fig. 6A), suggesting that PN neurons maintained molecularly distinct at P5. In addition, we identified the marker genes for each subtype by differential gene expression analysis (Fig. 6B, fig. S7A, and data S1). Notably, several PN subtypes at P5 share similar markers with those at E18.5 (figs. S6E and S7A), indicating that PN subtypes might be conserved between these two time points. Thus, we performed Friedman-Rafsky (FR) test to match the cell type in E18.5 and P5 datasets (*39*). We found concordant signatures between the two time points (Fig. 6C), suggesting that the PN neurons have partially acquired their molecular signatures by E18.5. Notably, we did not find a matched cell type for PN5 and PN6 in the E18.5 data (Fig. 6C). Given that PN5 and PN6 are two of the smallest populations among all subtypes, those two subtypes could be underrepresented in the E18.5 data due to the low total number of the PN neurons being sampled. Together, these data confirm that differentiated PN neurons are molecularly heterogeneous at both E18.5 and P5. Moreover, the preferential loss of ePN1 subtype at E18.5 in *Atoh1^S193A/−^* embryos spurred our interest in testing whether the six PN subtypes at P5 are affected by *Atoh1* hypomorphic mutation equally.

### The six molecularly defined PN subtypes are spatially segregated

**Fig. 7.**
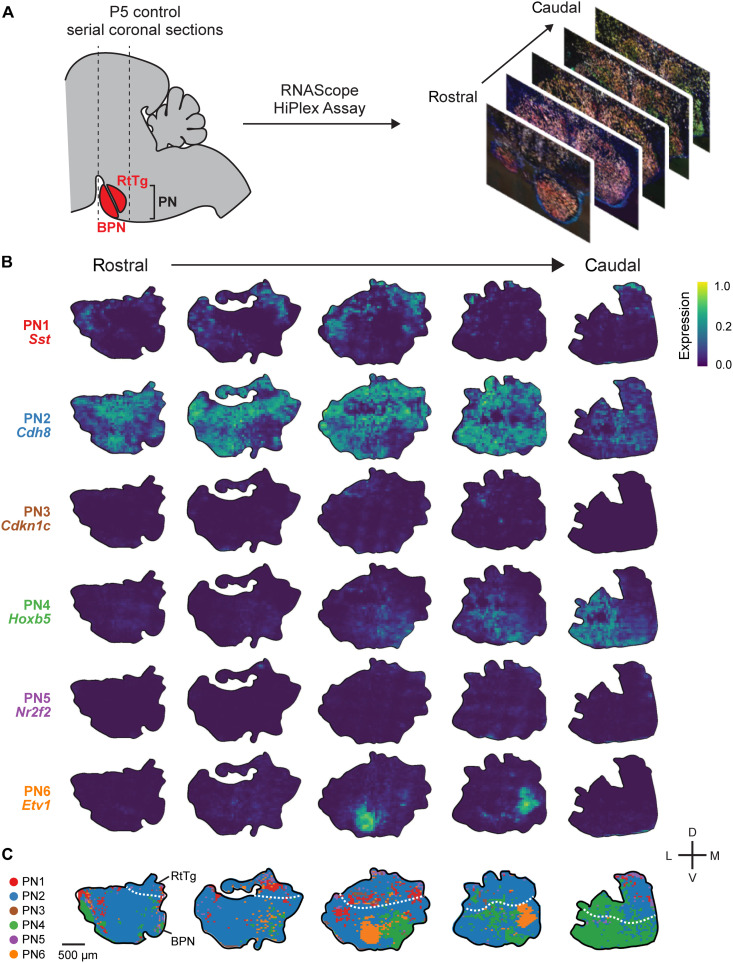
The spatial map of the PN subtypes in mice at P5. (**A**) Workflow of the RNAScope HiPlex Assay on P5 control mouse. (**B**) Expression patterns of the selective markers for each PN subtype across the rostral to caudal axis of the PN. (**C**) Schematic summary of the distribution of the PN subtypes across the PN. The dashed line denotes the border between RtTg and BPN.

We ultimately wanted to test whether the six PN subtypes were differentially compromised in the *Atoh1^S193A/−^* mice. However, examining the cellular phenotypes in PN subtypes is challenging without knowing where those subtypes are located. Unlike the well-characterized laminated structure in the cerebral cortex, cerebellum, and retina, the cellular organization of the PN has not been delineated. Thus, we first characterized the anatomic location of the six PN subtypes in control animals and then used the spatial information to identify the cellular phenotypes in *Atoh1^S193A/−^* animals. We performed fluorescence RNA in situ hybridization (ISH) with RNAScope HiPlex assay on serial coronal sections from control mouse brain at P5 (Fig. 7A). We used *TdTom* probes to label *Atoh1*-lineage neurons and define the region of interest (i.e., PN). By binning the images, we calculated the average expression of the marker genes within each bin across the PN (Fig. 7B). We found that all the markers exhibit spatial specificity except *Cdh8*. For example, *Hoxb5* is expressed in the caudal part of the PN, which is consistent with the previous study (*14*). Another marker, *Etv1*, is highly expressed in a restricted part of the medioventral PN. In contrast, *Cdh8* is globally expressed across PN, which is expected based on our P5 scRNA-seq data (Fig. 6B). Notably, we found that *Somatostatin* (*Sst*), a neuropeptide that is typically expressed in inhibitory neurons, is expressed in a subset of the PN neurons that exhibit a shell-like pattern at the rostral PN (Fig. 7B). To date, there are only few studies showing that *Sst* is coexpressed in subsets of excitatory neurons in pre-Bötzinger complex, lateral hypothalamus, and dorsolateral periaqueductal gray matter (*40*–*42*). Here, we demonstrated that *Sst* is coexpressed in a subset of *Slc17a6*^+^ [Vesicular Glutamate Transporter 2 (VGLUT2^+^)] PN neurons (fig. S7, B and C).

To find the corresponding PN subtypes between ISH and scRNA-seq data, we implemented *K*-nearest neighbor classification method to annotate the six PN subtypes on the representative regions of interest (Fig. 7C). PN neurons have been categorized into two nuclei, RtTg and BPN, based on their anatomic location. However, there has been no evidence showing that they are molecularly different. We found that PN6 is largely restricted to BPN, while PN3 and PN5 mostly reside at RtTg. In contrast, PN1, PN2, and PN4 are distributed across both nuclei. These findings suggest that RtTg and BPN have both shared and distinct neuronal subtypes. Together, our ISH data validate the expression of the marker genes identified by scRNA-seq and establish the spatial map of the PN subtypes that provides a higher resolution of the cytoarchitecture in the PN.

### PN subtypes have differential vulnerability to *Atoh1* hypomorphic mutation

**Fig. 8.**
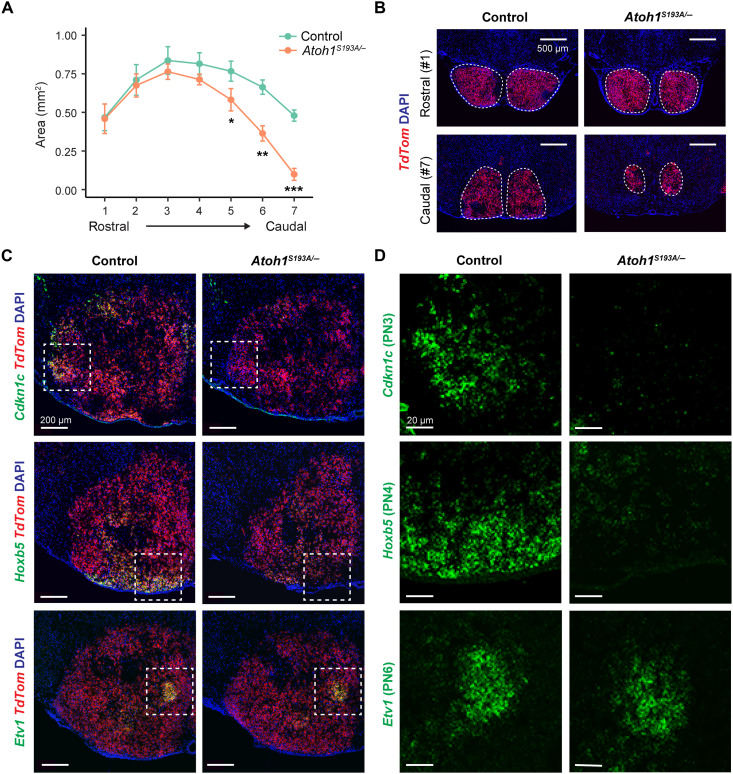
PN subtypes have different vulnerabilities to *Atoh1* hypomorphic mutation. (**A**) The size of the PN was determined by the areas across seven coronal sections (*n* = 3 per genotype). Data are presented as means ± SD. *P < 0.05; **P < 0.01; ***P < 0.001 by two-tailed unpaired *t* test. (**B**) Representative RNA ISH images for the most rostral (top) and the most caudal (bottom) sections from control (left) and *Atoh1^S193A/−^* (right) mice at P5. The PN neurons were labeled with *TdTom* probes. The nuclei were stained with DAPI. The dashed line encloses the area of the PN. Scale bars, 500 μm. (**C**) RNA ISH on control (left) and *Atoh1^S193A/−^* (right) mice at P5 using *Cdkn1c* (top), *Hoxb5* (middle), and *Etv1* (bottom) probes. PN neurons were labeled with *TdTom* probes. The nuclei were stained with DAPI. The box denotes the area shown in (D). Scale bars, 200 μm. (**D**) The zoom-in view of PN from the boxes in (C). The expression of *Cdkn1c* (top), *Hoxb5* (middle), and *Etv1* (bottom) were shown in green. Scale bars, 20 μm.

With the spatial map of the PN subtypes being established, we next performed fluorescence ISH to characterize PN subtypes in control and *Atoh1^S193A/−^* animals at P5. First, we used *TdTom* probes to identify PN across serial coronal sections. On the basis of the anatomic landmarks other than PN, we aligned the sections from different animals to proximity and quantified the size of PN in control and *Atoh1^S193A/−^* animals. Consistent with the whole-mount lacZ staining at P21 (Fig. 1D), we observed a reduced size of PN in *Atoh1^S193A/−^* animals at P5 (Fig. 8A). Moreover, we found that the caudal PN were most affected with 80% reduction at the most caudal section, while there is no significant difference at the rostral PN (Fig. 8, A and B).

Given that different PN subtypes exhibit spatial specificity along the rostral-caudal axis (Fig. 7C), we hypothesized that the PN subtypes are differentially affected in *Atoh1^S193A/−^* mice. We thus examined three PN subtypes (PN3, PN4, and PN6) in control and *Atoh1^S193A/−^* at P5 by performing dual ISH using *TdTom* probes and marker genes *Cdkn1c*, *Hoxb5*, and *Etv1*, respectively. *Cdkn1c* is the marker gene for PN3 subtype, which matches ePN1 subtype at E18.5 (Fig. 6C) and is predicted to be reduced in *Atoh1^S193A/−^* animals according to our E18.5 scRNA-seq data (Fig. 5D). Moreover, on the basis of the spatial map of the PN (Fig. 7C) and the selective loss of the caudal PN in *Atoh1^S193A/−^* mice (Fig. 8, A and B), we predicted that *Hoxb5*^+^ (PN4) was compromised by partial loss of function of *Atoh1*. Last, to test whether there is one subtype that is not sensitive to *Atoh1* hypomorphic mutation, we chose *Etv1*^+^ subtype (PN6). Given its specific location within the PN (Fig. 7C), changes in PN, if any, should be easily detected. We examined serial sections across the whole PN to exclude the possibility that certain subtype might be mislocated. We found fewer *Cdkn1c*^+^*TdTom*^+^ PN3 neurons in *Atoh1^S193A/−^* mice, accompanied by reduced size in the area where the Cdkn1c^+^ PN3 neurons are normally located in controls (Fig. 8, C and D, top). *Hoxb5*^+^*TdTom*^+^ PN4 neurons were also reduced in *Atoh1^S193A/−^* animals at the caudal sections (Fig. 8, C and D, middle). In contrast, *Etv1*^+^*TdTom*^+^ PN6 neurons were not affected in *Atoh1^S193A/−^* animals (Fig. 8, C and D, bottom). Together, these data demonstrated that although all PN subtypes were derived from *Atoh1*^+^ progenitors, certain PN subtypes such as PN3 and PN4 neurons were more vulnerable to partial loss of function of *Atoh1*, suggesting that the robustness of *Atoh1* function is critical for PN subtype cell fate decisions.

## DISCUSSION

In this study, we used a mouse model carrying an *Atoh1* hypomorphic mutation and implemented scRNA-seq technology to elucidate the roles of *Atoh1* in different cell states during PN development. We demonstrate that *Atoh1* is involved in multiple biological processes including regulating the cell cycle, differentiation, cell survival, migration, and the heterogeneity of the PN neurons. Moreover, we show that *Atoh1*-lineage PN neurons are classified as six subtypes based on their molecular signatures and provide a list of marker genes for future studies. PN subtypes display differential vulnerability to *Atoh1* hypomorphic mutation, which opens up questions such as how cell fate decisions are made and how perturbation of a transcription factor results in subtype-specific phenotypes.

### The interplay between *Atoh1* and Notch signaling

One interesting phenotype that we observed in *Atoh1^S193A/−^* mice was the marked increase in proliferating progenitors (Figs. 2F and 3B), suggesting that *Atoh1* might promote cell cycle exit. In addition, differential gene expression analysis revealed several dysregulated pathways including up-regulated Notch signaling in the proliferating progenitors (Fig. 4D). The interaction between *Atoh1* and Notch signaling has been demonstrated in the mammalian intestine (*43*, *44*). In the epithelium lining the crypts in the small intestine, the stem cells differentiate into secretory cells when they escape Notch activation by up-regulation of *Atoh1*. In contrast, the stem cells in which Notch is activated remain as progenitors at the crypts where *Wnt* is high. Here, we hypothesize that in cRL, partial loss of function of *Atoh1* leads to failure to escape Notch activation, which maintains the cells at the proliferating state. In line with this hypothesis, we observed up-regulation of the Notch receptor *Notch1* and its ligand *Dll1* as well as genes downstream from active Notch signaling including *Hes1* and *Hes5* in the proliferating progenitors of *Atoh1^S193A/−^* mice (data S2). Moreover, a recent study in zebrafish showed that inhibition of Notch activity at lower RL led to increased *atoh1b^+^* postmitotic precursors at the expense of *atoh1a^+^* proliferating progenitors (*45*). This study also supports our hypothesis that *Atoh1* and Notch antagonize each other at the cRL in mammals.

### The molecular heterogeneity of the PN neurons

One of the most puzzling questions in neurobiology is how to define a neuronal subtype. In the case of the PN, they have been considered as a group of heterogeneous neurons based on their anatomical location, origins at the cRL, and functions based on the cortico-ponto-cerebellar connectivity (*13*–*16*, *46*). However, whether the PN neurons can be further defined by their molecular signature has not been shown. scRNA-seq provides a powerful approach to classify the cell type unbiasedly based on transcription profiles. Here, we uncovered six PN subtypes in P5 mice (Fig. 6, A and B). These six PN subtypes were spatially segregated (Fig. 7), which raises an interesting question regarding whether this spatial map reflects the topographic connectivity between the cerebral cortex and the PN. For example, *Sst*^+^ PN subtype reside at the rostral dorsal part of the PN (Fig. 7B). This location coincides with where PN receive inputs from brain regions involved in visual pathway including visual cortex, superior colliculus, inferior colliculus, and pretectum (*47*). Whether there is a subtype-specific connectivity that also reflects the functionality needs further investigation. However, those studies cannot be done without genetic tools to target different PN subtypes. Our study identified and validated the marker genes for the six PN subtypes. In future studies, one can test whether different PN subtypes preferentially connect with specific groups of neurons in the cerebral cortex and/or cerebellum by intersectional labeling and viral tracing approaches with the molecular markers. In addition to the connectivity, the molecular markers that we identified can be used to manipulate PN neurons in a subtype-specific manner for functional characterization.

### From a pool of *Atoh1*^+^ progenitors to diverse PN subtypes

How does a pool of seemingly homogeneous progenitors give rise to a diverse population of neurons? In the case of *Atoh1*-lineage neurons in the hindbrain, *Atoh1*^+^ progenitors contribute to neurons in the cerebellum and dozens of brainstem nuclei depending on the rostrocaudal origins at RL and the timing of leaving from RL (*9*, *10*, *48*). PN, for example, were derived from rhombomere 6 to 8 where *Hox2* to *Hox5* are expressed (*10*). It has not been addressed, however, whether *Atoh1* contributes to the diversity of the subpopulation within one lineage (i.e., PN in this study). To this end, we tested whether PN subtypes display differential vulnerability to *Atoh1* hypomorphic mutation. On the basis of our scRNA-seq data at E18.5 and the histological analysis at P5, partial loss of function of *Atoh1* not only reduced the size of the PN but also reduced the diversity of the PN neurons by preferentially affecting PN3 and PN4 subtypes (Fig. 8, C and D). These data suggest that *Atoh1* may contribute to the acquisition or maintenance of the heterogeneity of the PN neurons.

In summary, this study dissected the functions of *Atoh1* during PN differentiation by characterizing the phenotypes in *Atoh1* hypomorphic mutant at single-cell resolution. Our data demonstrate that *Atoh1* regulates cell cycle exit, differentiation, migration, and survival during PN development and contributes to the diversity of the PN subtypes. In addition, this study also uncovers the molecular heterogeneity of the PN, which opens new doors for understanding the neural fate decisions, connectivity, and functionality of the PN neurons.

## MATERIALS AND METHODS

### Mice

The following mouse lines were used in this study: *Atoh1^lacZ/+^* (*23*), *Atoh1^S193A/+^* (*24*), *Atoh1^Cre/+^* (*28*), *Rosa^lsl-lacZ/+^* (JAX:02429), and *Rosa^lsl-TdTomato/+^* (JAX:007914). All mice were housed in a level 3, American Association for Laboratory Animal Science (AALAS)–certificated facility on a 14-hour light cycle. Husbandry, housing, euthanasia, and experimental guidelines were approved by the Institutional Animal Care & Use Committee (IACUC) at Baylor College of Medicine.

### Whole-mount X-galactosidase staining

The brains of P0 pups and P21 mice were dissected out in ice-cold phosphate-buffered saline (PBS). The samples were fixed in 4% paraformaldehyde (PFA) at 4°C for 1 hour (P0) or 2 hours (P21). After brief PBS wash at room temperature (RT), the samples were incubated in equilibration buffer [2 mM MgCl_2_, 0.05% sodium deoxycholate, 0.02% NP-40, and 0.1 M sodium phosphate (pH 7.3)] at 4°C for 15 min, followed by 2-hour incubation at 37°C in X-galactosidase (X-gal) reaction solution [X-gal (1 mg/ml), 5 mM potassium ferrocyanide, and 5 mM potassium ferricyanide in equilibration buffer]. After staining, the samples were washed with PBS three times at RT and fixed again in 4% PFA at 4°C for 1 hour (P0) or 4 hours (P21) before imaging.

### Sample preparation for scRNA-seq

The brains of embryos and P5 pups were dissected out in ice-cold HEBG medium (0.8× B27 and 0.25× GlutaMAX in hibernate E medium). For embryonic studies, hindbrains were collected from *Atoh1^S193A/−^* and its littermate control (one embryo per genotype, three independent replicates). For P5 study, PN were microdissected out and pooled from 11 to 13 pups (two independent replicates). Tissues were cut into small pieces and transferred to a 1.5-ml microcentrifuge tube using wide-bore pipette tips. The single-cell dissociation protocol was modified from the previous study (*49*). Briefly, the tissues were incubated with Worthington Papain solution at 37°C for 30 min at 800 rpm. At the end of incubation, the samples were transferred to a 15-ml falcon tube, followed by gentle trituration with serologic pipette. The cell pellets were collected by centrifuge at 200 rcf for 3 min at 4°C, washed, and resuspended in ice-cold sorting buffer (PBS with 0.05% fetal bovine serum). The single-cell resuspension was loaded to 30-μm cell drainer (CellTrics, SYSMEX 04-004-2326) to remove debris, followed by 4′,6-diamidino-2-phenylindole (DAPI) staining at RT for 5 min. TdTom^+^DAPI^−^ cells (120,000 to 150,000 cells per sample) were sorted into bovine serum albumin–coated 15-ml falcon tube by Sony SH800S cell sorter. The cells were pelleted by centrifuge at 200 rcf for 5 min at 4°C and resuspended in sorting buffer to make the final concentration of 1000 cells/μl.

### Library construction and sequencing

The cDNA libraries were constructed by 10X Genomics 3′ v3.1 kit following the user guide. Briefly, ~16,500 cells from one sample were mixed with reversed transcription master mix before loaded into Chromium Chip G. Droplets containing cells, reversed transcription reagents, and barcoded gel beads were generated by Chromium Controller. The first strand cDNA was amplified, fragmentated, and ligated with sequencing adaptors and sample indices. The cDNA libraries were sequenced by Illumina NovaSeq 6000.

### RNAScope HiPlex assay

The brain of the P5 pup was flash-frozen and embedded in optimal cutting temperature (OCT) compound. Coronal sections were made by cryostat (Leica) at 20 μm and store in −80°C until use. The RNAScope HiPlex assay (ACD, catalog no. 324419) was performed according to the manufacturer’s instructions. Briefly, sections were fixed in 4% PFA for 1 hour at RT, followed by dehydration with 50, 70, and 100% ethanol. The protease treatment was done by incubating the sections with protease III at RT for 30 min. Sections were hybridized with the 12 probes (catalog nos. 487941-T1, 845141-T2, 480301-T3, 557891-T4, 319171-T5, 458331-T6, 485461-T7, 503461-T8, 404631-T9, 503481-T10, 317041-T11, and 433411-T12) at 40°C for 2 hours, followed by three rounds of amplification. The TdTom reporter was helpful during sectioning to ensure that we covered the whole PN. However, it also introduced hazy background in HiPlex assay. Thus, we quenched the TdTom signal by incubating the sections with 5% formalin-fixed paraffin-embedded (FFPE) reagent (included in the kit) at RT for 30 min before developing the first round of the targets (T1 to T3). The nuclei were stained with DAPI. The 12 targets were detected in four rounds of imaging with three targets per round. Between each round, the fluorophores were cleaved and washed away before the next round of signal development. After image acquisition of all targets, the fluorophores were cleaved, and a blank image of each section was taken to serve as background images.

### Immunofluorescence staining

The heads of the E14.5 embryos were dissected in ice-cold PBS. After brief wash, the samples were fixed in 4% PFA at 4°C for 4 hours, followed by PBS wash and incubation in 30% sucrose in PBS at 4°C for 14 to 16 hours. The samples were cryopreserved and stored at −80°C until use. The coronal sections were collected on slides by cryostat (Leica) with 20 or 25 μm in thickness. The slides were rinsed with PBS to remove OCT, followed by permeabilization with 0.3% Triton X-100 in PBS for 15 min at RT. Antigen retrieval was performed by heating in antigen retrieval buffer [10 mM sodium citrate and 0.05% Tween-20 (pH 6.0)] at 85°C for 10 min [MKI67/red fluorescent protein (RFP) staining] or 30 min (CldU labeling). After blocking with blocking buffer (5% normal goat serum with 0.3% Triton X-100 in PBS) for 2 hours at RT, the sections were incubated with primary antibodies in blocking buffer at 4°C for 24 hours, followed by PBS wash three times. The sections were incubated with secondary antibodies in blocking buffer at RT for 2 hours. The counterstain was performed by DAPI staining at RT for 10 min. The slides were mounted with ProLong Gold mounting media (Invitrogen). The following antibodies were used in this study with the indicated dilutions: rat anti–5-bromo-2′-deoxyuridine (BrdU)/CldU (1:250; Abcam, ab6326), mouse anti-Ki67 (1:100; R&D Systems, AF7649), rabbit anti-RFP (1:2000; Rockland, 600-401-379), goat anti-rat Alexa Fluor 488 (1:500; Invitrogen, A-11006), goat anti-rabbit Alexa Fluor 555 (1:500; Invitrogen, A-21428), goat anti-mouse Alexa Fluor 647 (1:500; Invitrogen, A-21236).

### CldU labeling

CldU was prepared in sterile saline at 4.82 mg/ml and injected into pregnant dams intraperitoneally at E13.5 (85 mg/kg). Twenty-four hours later, the embryos were collected at E14.5, followed by immunofluorescence staining using rat anti-BrdU/CldU antibody (1:250; Abcam, ab2326).

### TUNEL assay

The sample preparation follows the same protocol that we used for immunofluorescence staining. Coronal sections were made by cryostat (Leica) at 25 μm. TUNEL assay was performed using DeadEnd Fluorometric TUNEL system (Promega) according to the user guide. Briefly, sections were fixed in 4% PFA at RT for 15 min, followed by proteinase K (20 μg/ml) treatment at RT for 12 min and a second fixation with 4% PFA for 5 min. The sections were equilibrated and incubated with fluorescein-labeled nucleotides and terminal deoxynucleotidyl transferase reaction mix at 37°C for 1 hour. After stopping the reaction by 2× saline-sodium citrate (SSC), the nuclei were stained with DAPI.

### Dual-color fluorescence RNA ISH

The brains from P5 pups were embedded in OCT, frozen on dry ice, and stored in −80°C until use. The sections were cut by cryostat (Leica) at 20 μm. We generated a digoxigenin (DIG)–labeled mRNA antisense probes against *Slc17a6* and *TdTom* and fluorescein isothiocyanate (FITC)–labeled mRNA against *Sst*, *Etv1*, and *Hoxb5* using reverse-transcribed mouse cDNA as template and DIG or FITC RNA labeling kits from Roche (Sigma-Aldrich). Primer sequences for *Sst*, *Slc17a6*, and *TdTom* probes are available in Allen Brain Atlas (www.brain-map.org). The following primers were used: 5′-ttcagaactcgggtctgctt-3′ and 5′-gaatcatgcaaaaggtggct-3′ for *Etv1* probe and 5′-gatggatctcagcgtcaacc-3′ and 5′-tatgagtctggctacagccg-3′ for *Hoxb5* probe. ISH was performed by the RNA In Situ Hybridization Core at Baylor College of Medicine using an automated robotic platform as previously described (*50*) with modifications of the protocol for double ISH. Briefly, two probes were hybridized to the tissue simultaneously. After the wash and blocking steps, the DIG-labeled probes were visualized by incubating with tyramide-Cy3 Plus (1:75; PerkinElmer) for 15 min. After washing in TNT (0.05% Tween-20 in 150mM NaCl and 100mM Tris-HCl, pH7.5), the remaining horseradish peroxidase (HRP) activity was quenched by a 10-min incubation in 0.2 M HCl. The sections were then washed in TNT and blocked in TNB for 15 min, followed by incubation with HRP-labeled sheep anti-FITC antibody (1:500 in TNB; Roche) at RT for 30 min. After washes in TNT, the FITC-labeled probes were visualized by incubating with tyramide-FITC Plus (1:75; PerkinElmer) for 15 min. Following washes in TNT, the slides were stained with DAPI (Invitrogen), washed again, removed from the machine, and mounted with ProLong Diamond (Invitrogen).

### Image acquisition

Images of the whole-mount tissues were obtained by Zeiss Axio Zoom.V16. All fluorescence images were obtained by Nikon Ti2E Inverted Motorized Microscope equipped with CSU-W1 Dual Camera-Dual Disk System with 405/488/561/640-nm lasers with 10×, 20×, or 40× objectives.

### Data preprocessing

The reads were aligned to customized genome that composed of mouse mm10 reference genome and woodchuck hepatitis virus post-transcriptional regulatory element (WPRE) sequence to ensure capturing the TdTom transcripts. The alignment and quantification of unique molecular identifier (UMI) were performed on 10× cloud analysis platform by CellRanger pipeline v5.0.1 with default parameters. Individual expression matrix of each sample was filtered by Seurat v4 package (*51*), followed by doublet removal using DoubletFinder v2 package (*52*). Briefly, cells with at least 1000 genes expressed and less than 1% of total UMIs that were mitochondrial genes were retained (see the code for detailed criteria depending on the age of the samples.) Doublets with high confidence score identified by DoubletFinder with default parameters were removed.

### Integration and clustering

The data integration and clustering were performed by Seurat package. The filtered datasets of the samples from the same age were combined by integration pipeline. Briefly, the filtered matrices were normalized and scaled by regressing out the percentage of mitochondrial genes with SCTransform (*53*). For E14.5 and E18.5 samples in which different genotypes were compared, a reference-based integration method was used by setting the first replicate as reference to cut down the computational power and time needed. For P5 study, the default integration method was used. Following the integration, principal components analysis (PCA) was performed. We selected the top 30 PCAs to generate the *K*-nearest neighbor graph, which was used to perform clustering with variated resolutions depending on the complexity of the dataset. To annotate the clusters, the top 10 genes that were highly expressed in each cluster were identified by FindAllMarkers function with default parameters.

### Trajectory analysis

The RNA assay of the Seurat object was extracted and transformed into SingleCellExperiment object (sce) by as.SingleCellExperiment function. The sce was used as input to infer the trajectory by slingshot (*30*). Kolmogorov-Smirnov test was used to assess whether the distribution of pseudo-time is identical between the genotypes.

### Differential expression analysis

We performed differential expression analysis between control and *Atoh1^S193A/−^* samples for each cell state by FindMarkers function from Seurat package. Only genes that were expressed in at least 10% of the cells were included. MAST (*54*) was used as test method. The *P* value was corrected by Benjamini-Hochberg FDR method.

### GO analysis

GO analysis was performed using a web-based tool called g:Profiler (*55*) with custom statistic domain scope. The up- and down-regulated DEGs (log_2_FC > 0.25 and FDR < 0.05) within progenitors, intermediate progenitors, and migrating neurons-1 were used as inputs independently. GO biological process was selected for the analysis.

### Image processing for RNAScope HiPlex assay

Five confocal z-stacks of images were collected at 100- and 200-μm intervals along the rostral-caudal axis of the pontine nucleus for each of the nine transcripts. In addition, DAPI images were also collected to serve as reference images. Individual z-stacks consisted of 10 images separated by 0.9 μm. The z-stacks were first processed by taking the max intensity projection and cropping the resulting transcript image such that the midline of the pontine aligned with the right-hand border of each image.

### Visualizing transcript expression

Overlaying the transcript images onto the DAPI reference images (*56*) revealed that some transcripts were not consistently colocalized to the nucleus. To address this ambiguity in our analysis, we did not associate transcript expression with individual cells. Rather, we binned each image into a set of tiles measuring 34 μm × 31 μm and measured the transcript expression in each tile (*57*). Since transcripts may be expressed at very different levels, we normalized the expression for each transcript by the max expression recorded across the five image locations in the pontine. Thus, in Fig. 7B, the expression for each transcript varies from 0 to 1 across the five rostral to caudal sections. To identify the boundaries of the pontine, the max intensity projection across all the transcripts at a given imaging location was smoothed with a Gaussian filter with an SD of 35 μm. This filtered image was converted to a binary image by mean thresholding, and any remaining holes were morphologically closed using a 5-μm × 5-μm structuring element. Last, the pontine boundary was taken to be the edge of the largest single region in the closed binary image.

### Quantification of the immunostaining and statistics

The cell number of the CldU^+^TdTom^+^ (Fig. 3F) and TUNEL^+^ (Fig. 4F) was determined by manual counting on imageJ. The percentage of MKI67 signal overlapping with TdTom (Fig. 3C) was calculated based on Manders’ coefficients using JACoP (*58*). For the statistical test, we used mixed-model analysis of variance (ANOVA) to compare genotype using *lme4* package on R (*59*) to count for variations among technical and biological replicates. The person who performed the quantification was blinded to the genotypes of the samples.

### Classification of RNA ISH transcripts

The transcript expression levels for each tile in the ISH images can be represented as a nine-dimensional vector, one component for each transcript. To classify these transcript expression vectors as one of the six classes determined from the single-cell RNA-seq analysis, we built a *K*-nearest neighbor classifier (*60*). Specifically, for each cell (*n* = 7029) from the RNA-seq clustering results, we extracted the same transcripts as measured in the ISH and the cluster identity. Each of these nine-component vectors was normalized (*60*) and, along with their respective cluster IDs, was used to train and validate our classifier. The *K*-nearest neighbor classifier measures the distance between each nine-component vector and a preset number of neighbor vectors to predict class membership. To determine the number of neighbors, we performed sixfold cross-validation on the training dataset and found that 25 neighbors provided an average test accuracy of 80%. We then used this 25 nearest neighbor model to predict the cluster identity of each tile in the ISH images. The border of the RtTg and BPN in Fig. 7C was defined using the reference atlas of P6 mouse brain (*61*).

## Supplementary Materials

### This PDF file includes:

Figs. S1 to S7 Table S1 Legends for data S1 to S3 References

## Notes

View/request a protocol for this paper from *Bio-protocol*.
