# Supplementary material for: Atoh1 drives the heterogeneity of the pontine nuclei neurons and promotes their differentiation
Source: Sci Adv. 2023 Jun 30;9(26):eadg1671. doi: 10.1126/sciadv.adg1671 (PMC10313176; doi:10.1126/sciadv.adg1671)
Supplement: Supplementary file 1 — Figs. S1 to S7 Table S1 Legends for data S1 to S3 References [file sciadv.adg1671_sm.pdf]

Science Advances
AAAS

Supplementary Materials for

Atoh1 drives the heterogeneity of the pontine nuclei neurons and promotes their differentiation

Sih-Rong Wu *et al.*

Corresponding author: Huda Y. Zoghbi, hzoghbi@bcm.edu

Sci. Adv. **9**, eadg1671 (2023)
DOI: 10.1126/sciadv.adg1671

The PDF file includes:

Figs. S1 to S7
Table S1
Legends for data S1 to S3
References

Other Supplementary Material for this manuscript includes the following:

Data S1 to S3

Supplementary Figures and legends

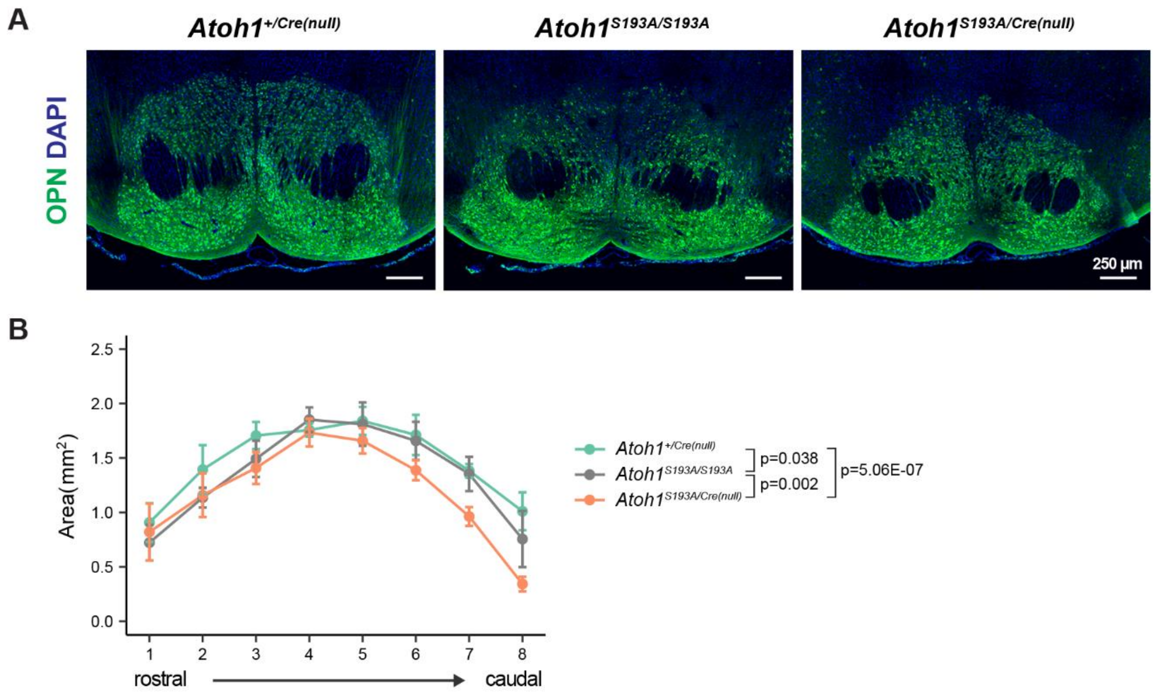

Fig. S1. The size of PN was reduced in *Atoh1$^{S193A/S193A}$* and *Atoh1$^{S193A/-}$* mice at P21.

(**A**) Immunofluorescence staining of osteopontin (OPN) on coronal section of mouse P21 brains. The nuclei were stained with DAPI. Scale bar, 250 µm. (**B**) The size of the PN was determined by OPN^{+} PN neurons across eight coronal sections (n = 3 per genotype). Two-Way ANOVA followed by Tukey HSD post hoc test.

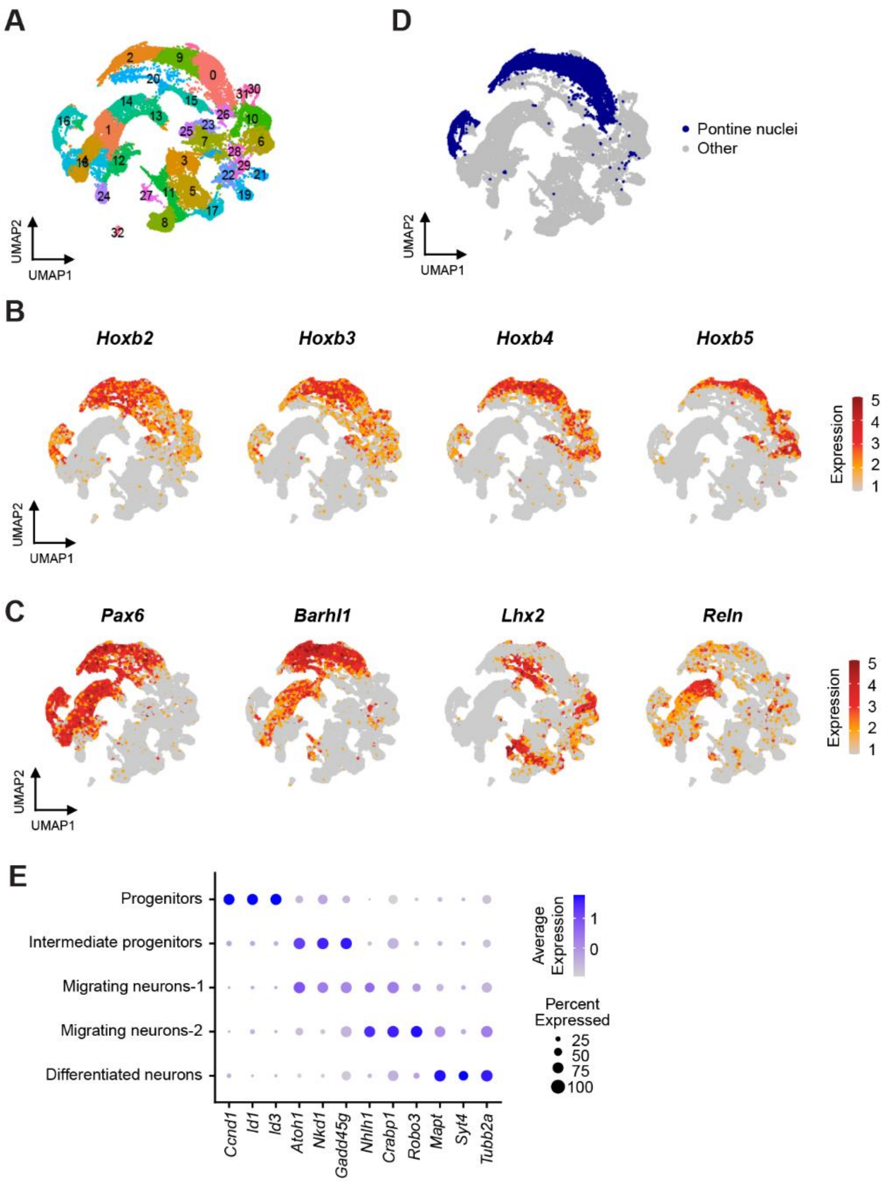

Fig. S2. Markers for pontine nuclei subset and individual cell state at E14.5.

(**A**) UMAP representation of scRNA-seq data of E14.5 hindbrains. (**B**) and (**C**) Expression patterns of the selective markers to identify PN subset. *Hoxb2-Hoxb5* are markers for cRL-derived *Atoh1*-lineage (cluster 16, 2, 9, 0, 20, 15, 26, 23, 28, 6, 10, 30, 31). *Pax6* and *Barhl1* are markers for both PN and cerebellar EGL so we excluded EGL using EGL markers *Lhx2* and *Reln* (cluster 20 and 15). Cluster 26, 23, 28, 6, 10, 30, and 31 were also excluded for not expressing *Pax6* and *Barhl1*. (**D**) UMAP representation of scRNA-seq data of E14.5 hindbrains with PN subset highlighted in blue. The PN subset was determined using the

combination expression of the markers shown in (B) and (C). (**E**) Dot plot of the selective markers for developing PN at E14.5. Three markers were presented for each cell state.

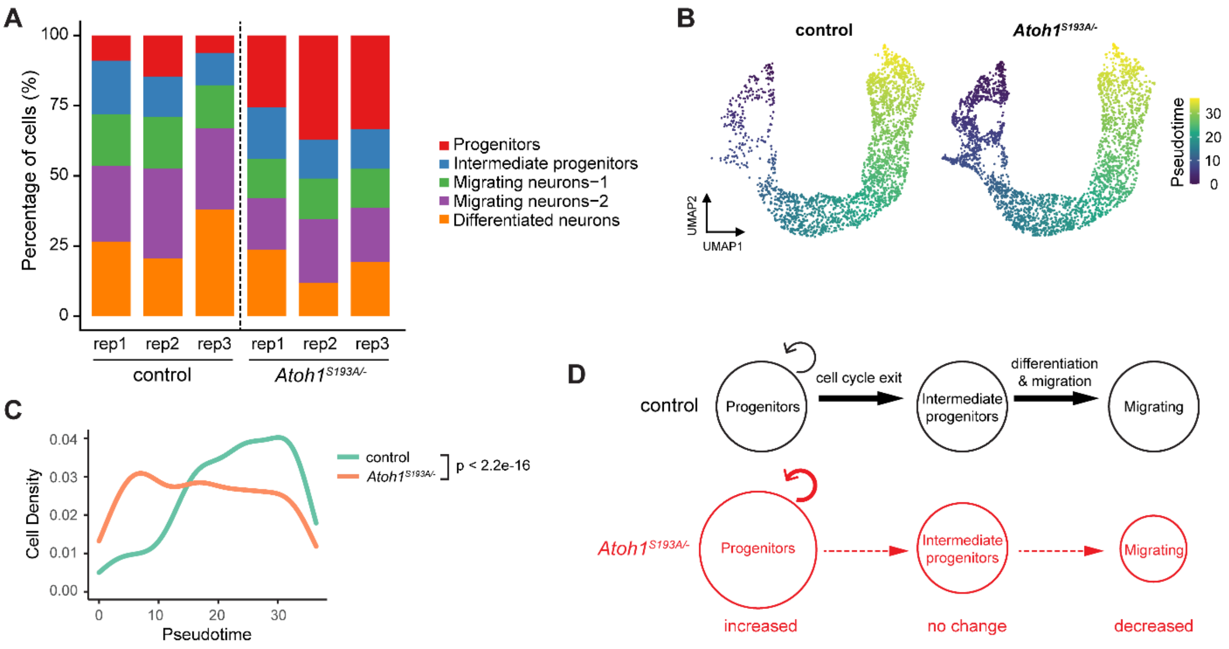

Fig. S3. Trajectory analysis of the PN scRNA-seq data at E14.5.

(**A**) Proportion of the cells in each cell state. The data of individual sample are shown. (**B**) Pseudotime analysis on control (left) and *Atoh1*$^{S193A/-}$ (right) animals represented on UMAP. (**C**) The cell density along pseudotime of control and *Atoh1*$^{S193A/-}$ animals. p < 2.2e-16 by progression test (see methods). (**D**) The mechanisms underlying the altered proportion of the cells in each cell state in *Atoh1*$^{S193A/-}$ mice. The thickness of the arrows denotes the strength of the indicated biological process. The size of the circle represents the proportion of the cells in each cell state.

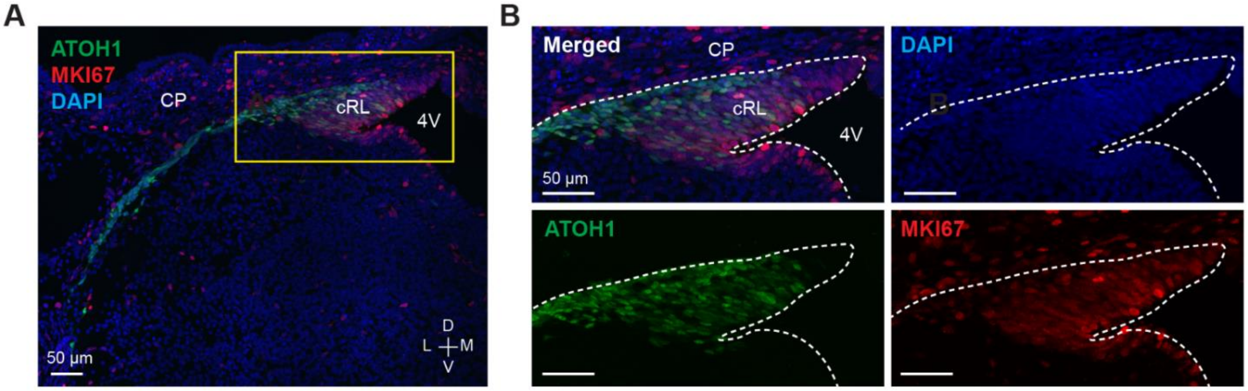

Fig. S4. Proliferating progenitors and intermediate progenitors are located at medial and lateral cRL, respectively.

(**A**) Immunostaining of GFP and MKI67 on *Atoh1*$^{Cre/GFP}$*; Rosa*$^{lsl\text{-}tdTom/+}$ mice (*62*) at E14.5. Anti-GFP antibody was used to detect ATOH1-GFP fusion protein expression. Anti-MKI67 antibody was used to label proliferating cells. The nuclei were stained with DAPI. The representative image of coronal section is shown. The yellow box denotes the cropped region in (B). CP, choroid plexus; cRL, caudal rhombic lip; 4V. fourth ventricle; L, lateral; M, medial; D, dorsal; V, ventral. Scale bar, 50 µm. (**B**) Zoom-in view of the box in (A) with split channels. The dashed line indicates the outline of the cRL. Scale bar, 50 µm.

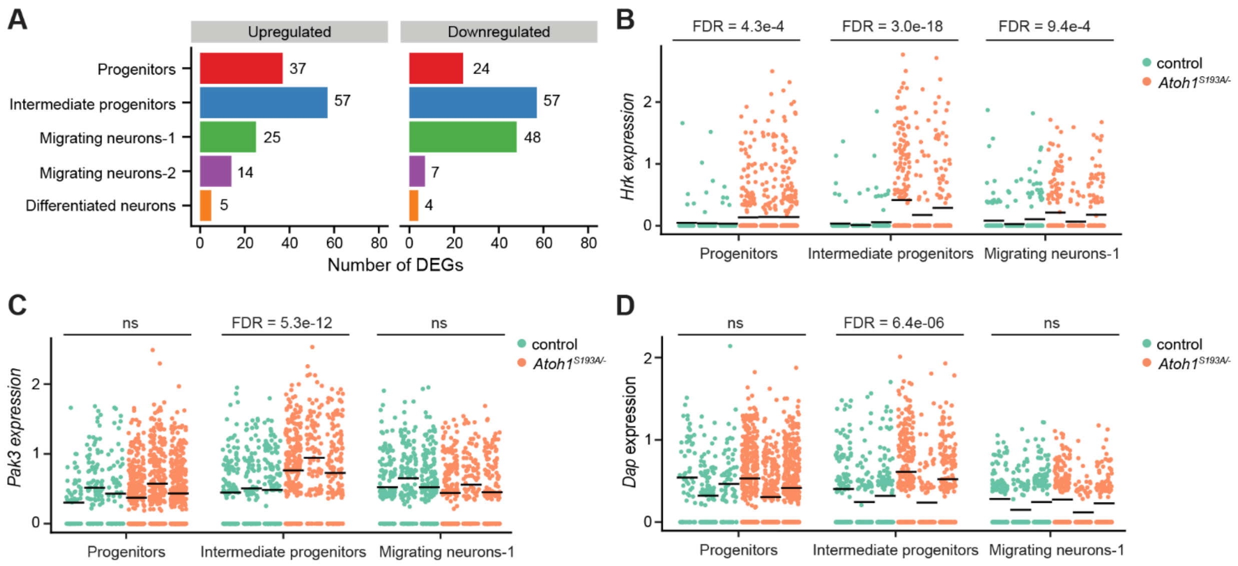

Fig. S5. Differential gene expression analysis of the PN scRNA-seq data at E14.5.

(**A**) Numbers of the upregulated (left) and downregulated (right) DEGs in each cell state. The DEGs were calculated by downsampling to match an equal number of the cells being analyzed in each cell state. The cutoff for DEG is $\log_2 FC > 0.25$ and FDR < 0.05. (**B**) to (**D**) Normalized expression levels of *Hrk* (B), *Pak3* (C), and *Dap* (D) in progenitors, intermediate progenitors, and migrating neurons-1. The dots represent individual cells. The expression level for individual sample was shown and grouped by genotype. The horizontal line denotes the mean expression level in each genotype.

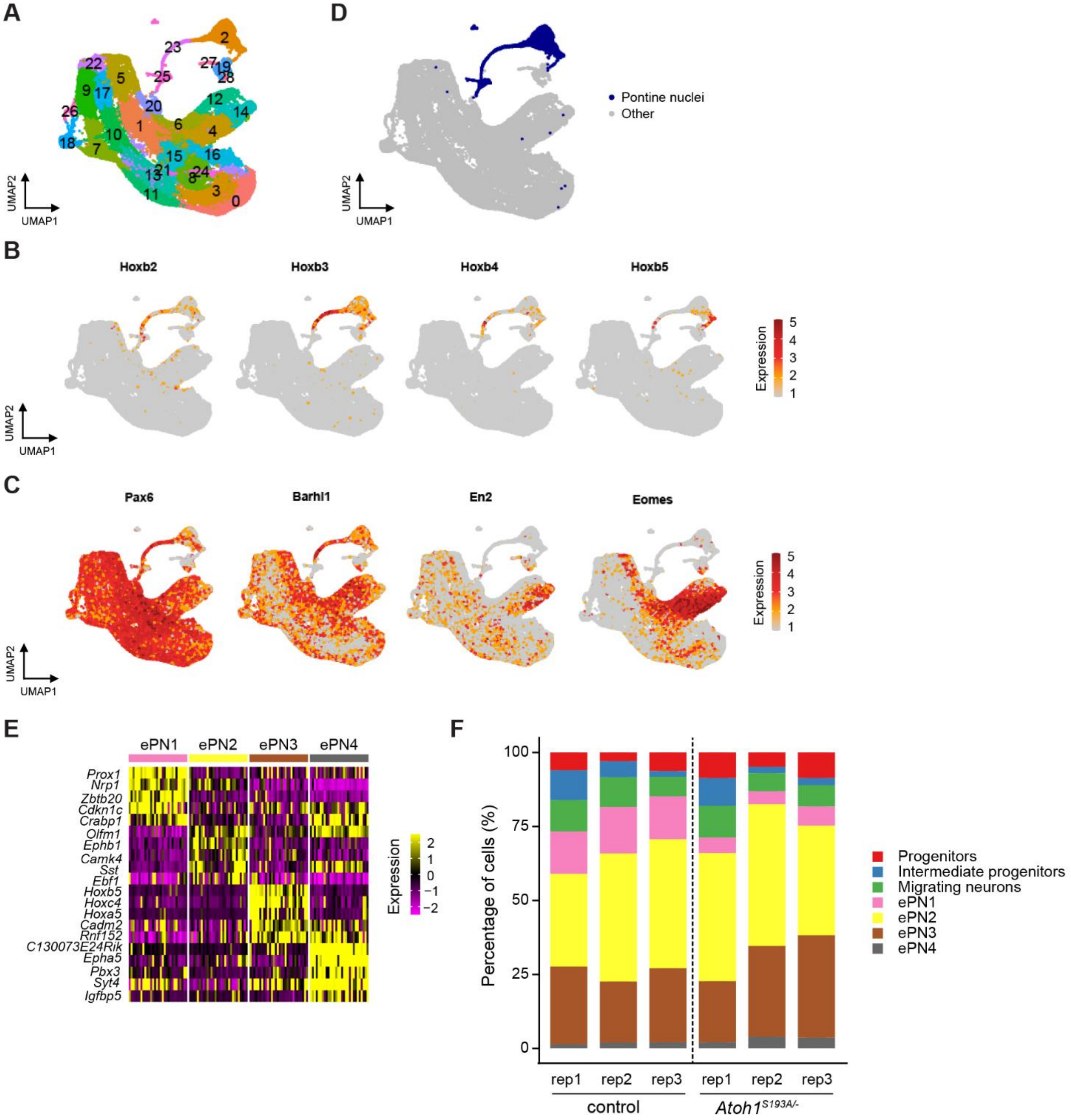

Fig. S6. Markers for pontine nuclei subset and individual ePN subtype at E18.5.

(**A**) UMAP representation of scRNA-seq data of E18.5 hindbrains. (**B**) and (**C**) Expression patterns of the selective markers to identify PN subset. *Hoxb2*-*Hoxb5* are markers for cRL-derived *Atoh1*-lineage (cluster 25, 23, 2, 27, 19, 28). *Pax6* and *Barhl1* are markers for PN and EGL (cluster 23, 2, 19). We exclude cluster 19 using *En2* and *Eomes*, markers for rRL-derived *Atoh1*-lineage. (**D**) UMAP representation of scRNA-seq

data of E18.5 hindbrains with PN subset highlighted in blue. The PN subset was determined using the combination expression of the markers shown in (B) and (C). (**E**) Heatmap of the top 5 markers for embryonic PN (ePN) subtypes at E18.5. (F) Proportion of the cells in each cell state. The data of individual sample are shown.

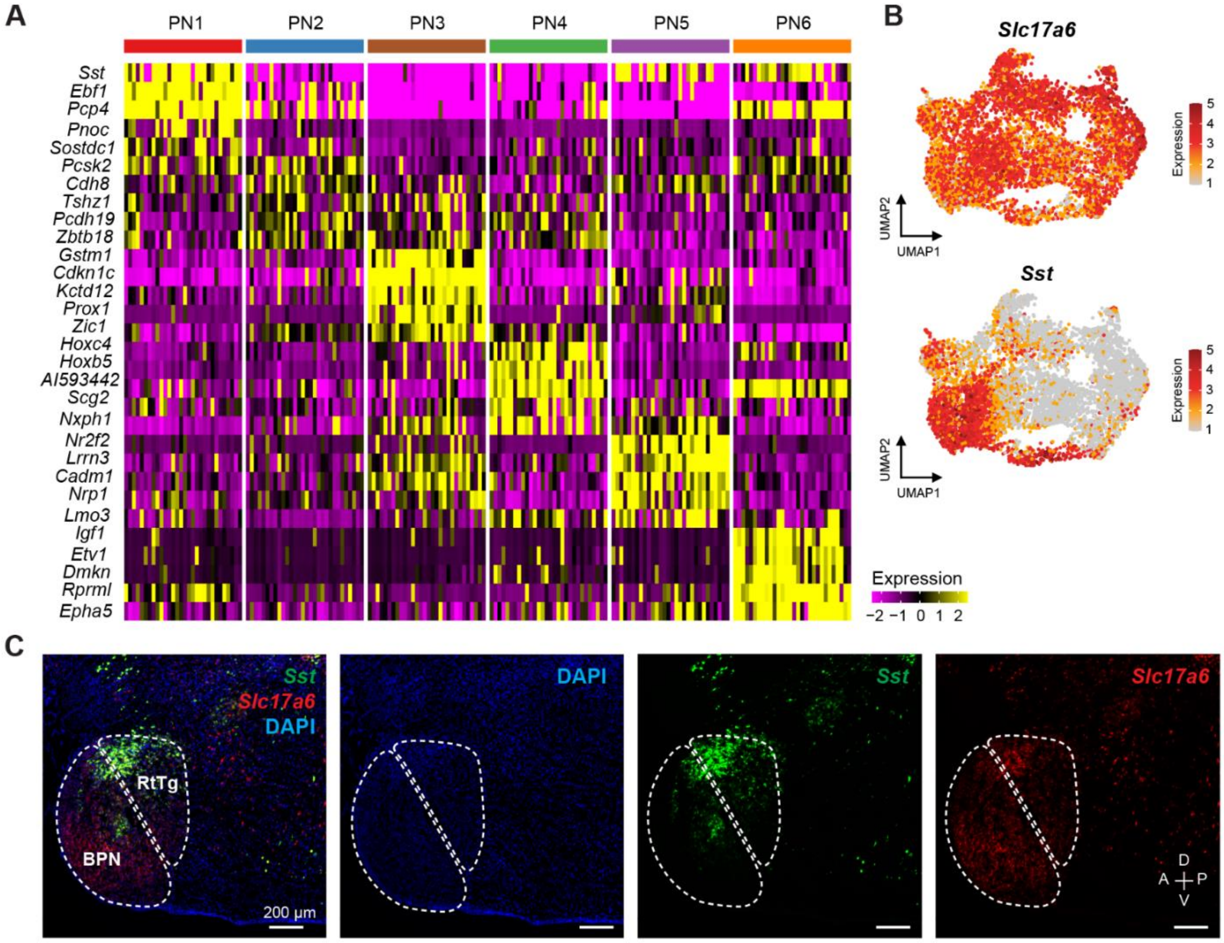

Fig. S7. Top 5 markers for the six PN subtypes at P5 and validation with FISH.

(**A**) Heatmap of the top 5 markers for six PN subtypes at P5. (**B**) The expression levels of *Slc17a6* and *Sst* visualized on UMAP of the scRNA-seq data of P5 PN. (**C**) Dual RNA *in situ* hybridization of *Sst* and *Slc17a6* (*vglut2*) on the P5 control mouse brain. The representative image of the sagittal section is shown. The nuclei were stained with DAPI. The dashed line denotes the outline of the PN including reticulotegmental nucleus (RtTg) and basal pontine nucleus (BPN). A, anterior; P, posterior; D, dorsal; V, ventral. Scale bar, 200 µm.

SampleID	Timepoint	Genotype	Sex	Region	Replicate	TotalCellNumber	MeanReadsPerCell	MedianGenesPerCell	MedianUMIperCell	TotalGenes
01	E14.5	control	F	Hindbrain	1	7,983	63,753	4,298	16,086	22,962
02	E14.5	*Atoh1*$^{S193A/-}$	M	Hindbrain	1	9,672	60,750	4,394	16,668	23,343
03	E14.5	control	M	Hindbrain	2	10,406	78,898	4,290	15,786	22,890
04	E14.5	*Atoh1*$^{S193A/-}$	M	Hindbrain	2	9,000	88,699	4,551	17,034	23,040
05	E14.5	control	F	Hindbrain	3	12,869	63,394	3,955	14,225	23,389
06	E14.5	*Atoh1*$^{S193A/-}$	F	Hindbrain	3	10,812	96,854	4,623	18,507	23,661
07	E18.5	control	M	Hindbrain	1	9,587	106,086	4,422	15,305	22,771
08	E18.5	*Atoh1*$^{S193A/-}$	M	Hindbrain	1	13,021	76,909	4,125	13,356	23,199
09	E18.5	control	M	Hindbrain	2	10,565	75,301	3,723	11,683	22,484
10	E18.5	*Atoh1*$^{S193A/-}$	M	Hindbrain	2	9,292	80,665	3,835	12,255	22,208
11	E18.5	control	F	Hindbrain	3	11,200	93,336	3,980	13,175	23,328
12	E18.5	*Atoh1*$^{S193A/-}$	F	Hindbrain	3	10,384	51,111	3,446	9,837	22,685
13	P5	control	Mix*	Pontine nuclei	1	15,821	107,415	3,096	8,433	24,220
14	P5	control	Mix*	Pontine nuclei	2	9,586	125,723	3,198	9,835	23,597

* The sample was pooled from 11-13 embryos.

Table. S1. Sample information and quality control for scRNA-seq data.

Data S1. The top 10 markers and the full list of markers for different timepoints.

Data S2. Differentially expressed genes (FDR < 0.05) between the control and *Atoh1*$^{S193A/-}$ mice from E14.5 PN scRNA-seq data.

Data S3. Gene ontology enrichment analysis of the differentially expressed genes between control and *Atoh1*$^{S193A/-}$ mice grouped by cell state.

REFERENCES AND NOTES

1. E. V. Evarts, W. T. Thach, Motor mechanisms of the CNS: Cerebrocerebellar interrelations. *Annu. Rev. Physiol.* **31**, 451–498 (1969).

2. C. R. Legg, B. Mercier, M. Glickstein, Corticopontine projection in the rat: The distribution of labelled cortical cells after large injections of horseradish peroxidase in the pontine nuclei. *J. Comp. Neurol.* **286**, 427–441 (1989).

3. P. Brodal, J. G. Bjaalie, Organization of the pontine nuclei. *Neurosci. Res.* **13**, 83–118 (1992).

4. C. Schwarz, P. Thier, Binding of signals relevant for action: Towards a hypothesis of the functional role of the pontine nuclei. *Trends Neurosci.* **22**, 443–451 (1999).

5. J. D. Schmahmann, R. Ko, J. MacMore, The human basis pontis: Motor syndromes and topographic organization. *Brain* **127**, 1269–1291 (2004).

6. K. Tziridis, P. W. Dicke, P. Thier, Pontine reference frames for the sensory guidance of movement. *Cereb. Cortex* **22**, 345–362 (2012).

7. G.-Y. Wu, S.-L. Liu, J. Yao, L. Sun, B. Wu, Y. Yang, X. Li, Q.-Q. Sun, H. Feng, J.-F. Sui, Medial prefrontal cortex-pontine nuclei projections modulate suboptimal cue-induced associative motor learning. *Cereb. Cortex* **28**, 880–893 (2018).

8. C. I. Rodriguez, S. M. Dymecki, Origin of the precerebellar system. *Neuron* **27**, 475–486 (2000).

9. V. Y. Wang, M. F. Rose, H. Y. Zoghbi, Math1 expression redefines the rhombic lip derivatives and reveals novel lineages within the brainstem and cerebellum. *Neuron* **48**, 31–43 (2005).

10. A. F. Farago, R. B. Awatramani, S. M. Dymecki, Assembly of the brainstem cochlear nuclear complex is revealed by intersectional and subtractive genetic fate maps. *Neuron* **50**, 205–218 (2006).

11. J. Altman, S. A. Bayer, Development of the precerebellar nuclei in the rat: IV. The anterior precerebellar extramural migratory stream and the nucleus reticularis tegmenti pontis and the basal pontine gray. *J. Comp. Neurol.* **257**, 529–-552 (1987).

12. T. Okada, K. Keino-Masu, M. Masu, Migration and nucleogenesis of mouse precerebellar neurons visualized by in utero electroporation of a green fluorescent protein gene. *Neurosci. Res.* **57**, 40–49 (2007).

13. A. Brodal, J. Jansen, The ponto-cerebellar projection in the rabbit and cat; experimental investigations. *J. Comp. Neurol.* **84**, 31–118 (1946).

14. T. D. Meglio, C. F. Kratochwil, N. Vilain, A. Loche, A. Vitobello, K. Yonehara, S. M. Hrycaj, B. Roska, A. H. F. M. Peters, A. Eichmann, D. Wellik, S. Ducret, F. M. Rijli, Ezh2 orchestrates topographic migration and connectivity of mouse precerebellar neurons. *Science* **339**, 204–207 (2013).

15. T. B. Leergaard, K. A. Lyngstad, J. H. Thompson, S. Taeymans, B. P. Vos, E. de Schutter, J. M. Bower, J. G. Bjaalie, Rat somatosensory cerebropontocerebellar pathways: Spatial relationships of the somatotopic map of the primary somatosensory cortex are preserved in a three-dimensional clustered pontine map. *J. Comp. Neurol.* **422**, 246–266 (2000).

16. J. U. Henschke, J. M. Pakan, Disynaptic cerebrocerebellar pathways originating from multiple functionally distinct cortical areas. *eLife* **9**, e59148 (2020).

17. N. A. Bermingham, B. A. Hassan, V. Y. Wang, M. Fernandez, S. Banfi, H. J. Bellen, B. Fritzsch, H. Y. Zoghbi, Proprioceptor pathway development is dependent on Math1. *Neuron* **30**, 411–422 (2001).

18. N. A. Bermingham, B. A. Hassan, S. D. Price, M. A. Vollrath, N. Ben-Arie, R. A. Eatock, H. J. Bellen, A. Lysakowski, H. Y. Zoghbi, Math1: An essential gene for the generation of inner ear hair cells. *Science* **284**, 1837–1841 (1999).

19. S. M. Maricich, S. A. Wellnitz, A. M. Nelson, D. R. Lesniak, G. J. Gerling, E. A. Lumpkin, H. Y. Zoghbi, Merkel cells are essential for light-touch responses. *Science* **324**, 1580–1582 (2009).

20. N. F. Shroyer, M. A. Helmrath, V. Y.-C. Wang, B. Antalffy, S. J. Henning, H. Y. Zoghbi, Intestine-specific ablation of mouse atonal homolog 1 (Math1) reveals a role in cellular homeostasis. *Gastroenterology* **132**, 2478–2488 (2007).

21. M. F. Rose, K. A. Ahmad, C. Thaller, H. Y. Zoghbi, Excitatory neurons of the proprioceptive,

interoceptive, and arousal hindbrain networks share a developmental requirement for Math1. *Proc. Natl. Acad. Sci. U.S.A.* **106**, 22462–22467 (2009).

22. T. Višnjar, A. Maver, K. Writzl, O. Maloku, G. Bergant, H. Jaklič, D. Neubauer, F. Fogolari, N. P. Meglič, B. Peterlin, Biallelic *ATOH1* gene variant in siblings with pontocerebellar hypoplasia, developmental delay, and hearing loss. *Neurol. Genet.* **8**, e677 (2022).

23. N. Ben-Arie, B. A. Hassan, N. A. Bermingham, D. M. Malicki, D. Armstrong, M. Matzuk, H. J. Bellen, H. Y. Zoghbi, Functional conservation of *atonal* and *Math1*in the CNS and PNS. *Development* **127**, 1039–1048 (2000).

24. W. R. Xie, H.-I. Jen, M. L. Seymour, S.-Y. Yeh, F. A. Pereira, A. K. Groves, T. J. Klisch, H. Y. Zoghbi, An Atoh1-S193A phospho-mutant allele causes hearing deficits and motor impairment. *J. Neurosci.* **37**, 8583–8594 (2017).

25. X. Jin, S. K. Simmons, A. Guo, A. S. Shetty, M. Ko, L. Nguyen, V. Jokhi, E. Robinson, P. Oyler, N. Curry, G. Deangeli, S. Lodato, J. Z. Levin, A. Regev, F. Zhang, P. Arlotta, *In vivo* perturb-seq reveals neuronal and glial abnormalities associated with autism risk genes. *Science* **370**, eaaz6063 (2020).

26. I. Schaffner, M.-T. Wittmann, T. Vogel, D. C. Lie, Differential vulnerability of adult neurogenic niches to dosage of the neurodevelopmental-disorder linked gene Foxg1. *Mol. Psychiatry*, **28**, 497–514 (2023).

27. P. Soriano, Generalized lacZ expression with the ROSA26 Cre reporter strain. *Nat. Genet.* **21**, 70–71 (1999).

28. H. Yang, X. Xie, M. Deng, X. Chen, L. Gan, Generation and characterization of Atoh1-Cre knock-in mouse line. *Genesis* **48**, 407–413 (2010).

29. B. Phipson, C. B. Sim, E. R. Porrello, A. W. Hewitt, J. Powell, A. Oshlack, *Propeller*: Testing for differences in cell type proportions in single cell data. *Bioinformatics* **38**, 4720–4726 (2022).

30. K. Street, D. Risso, R. B. Fletcher, D. Das, J. Ngai, N. Yosef, E. Purdom, S. Dudoit, Slingshot: Cell lineage and pseudotime inference for single-cell transcriptomics. *BMC Genomics* **19**, 477 (2018).

31. H. R. de Bézieux, K. Van den Berge, K. Street, S. Dudoit, Trajectory inference across multiple conditions with condiments: Differential topology, progression, differentiation, and expression. bioRxiv 2021.03.09.433671 [Preprint]. 10 March 2021. https://doi.org/10.1101/2021.03.09.433671.

32. T. J. Klisch, Y. Xi, A. Flora, L. Wang, W. Li, H. Y. Zoghbi, In vivo *Atoh1* targetome reveals how a proneural transcription factor regulates cerebellar development. *Proc. Natl. Acad. Sci. U.S.A.* **108**, 3288–3293 (2011).

33. A. W. Helms, A. L. Abney, N. Ben-Arie, H. Y. Zoghbi, J. E. Johnson, Autoregulation and multiple enhancers control Math1 expression in the developing nervous system. *Development* **127**, 1185–1196 (2000).

34. S. Li, F. Qiu, A. Xu, S. M. Price, M. Xiang, Barhl1 regulates migration and survival of cerebellar granule cells by controlling expression of the neurotrophin-3 gene. *J. Neurosci.* **24**, 3104–3114 (2004).

35. T. Schmid, M. Kruger, T. Braun, NSCL-1 and -2 control the formation of precerebellar nuclei by orchestrating the migration of neuronal precursor cells. *J. Neurochem.* **102**, 2061–2072 (2007).

36. T. Cai, H.-I. Jen, H. Kang, T. J. Klisch, H. Y. Zoghbi, A. K. Groves, Characterization of the transcriptome of nascent hair cells and identification of direct targets of the Atoh1 transcription factor. *J. Neurosci.* **35**, 5870–5883 (2015).

37. H. V. Yu, L. Tao, J. Llamas, X. Wang, J. D. Nguyen, T. Trecek, N. Segil, POU4F3 pioneer activity enables ATOH1 to drive diverse mechanoreceptor differentiation through a feed-forward epigenetic mechanism. *Proc. Natl. Acad. Sci. U.S.A.* **118**, e2105137118 (2021).

38. H. C. Lai, T. J. Klisch, R. Roberts, H. Y. Zoghbi, J. E. Johnson, *In vivo* neuronal subtype-specific targets of Atoh1 (Math1) in dorsal spinal cord. *J. Neurosci.* **31**, 10859–10871 (2011).

39. Y. Zhang, B. Aevermann, R. Gala, R. H. Scheuermann, Cell type matching in single-cell RNA-sequencing data using FR-match. *Sci. Rep.* **12**, 9996 (2022).

40. R. L. Stornetta, D. L. Rosin, H. Wang, C. P. Sevigny, M. C. Weston, P. G. Guyenet, A group of glutamatergic interneurons expressing high levels of both neurokinin-1 receptors and somatostatin

identifies the region of the pre-Bötzinger complex. *J. Comp. Neurol.* **455**, 499–512 (2003).

41. L. E. Mickelsen, M. Bolisetty, B. R. Chimileski, A. Fujita, E. J. Beltrami, J. T. Costanzo, J. R. Naparstek, P. Robson, A. C. Jackson, Single-cell transcriptomic analysis of the lateral hypothalamic area reveals molecularly distinct populations of inhibitory and excitatory neurons. *Nat. Neurosci.* **22**, 642–656 (2019).

42. N. Winke, F. Aby, D. Jercog, G. Zoé, D. Girard, M. Landry, L. Castell, E. Valjent, S. Valerio, P. Fossat, C. Herry, Brainstem somatostatin-expressing cells control the emotional regulation of pain behavior. bioRxiv 2022.01.20.476899 [Preprint]. 22 January 2022. https://doi.org/10.1101/2022.01.20.476899.

43. Q. Yang, N. A. Bermingham, M. J. Finegold, H. Y. Zoghbi, Requirement of Math1 for secretory cell lineage commitment in the mouse intestine. *Science* **294**, 2155–2158 (2001).

44. R. Sancho, C. A. Cremona, A. Behrens, Stem cell and progenitor fate in the mammalian intestine: Notch and lateral inhibition in homeostasis and disease. *EMBO Rep.* **16**, 571–581 (2015).

45. I. Belzunce, C. Belmonte-Mateos, C. Pujades, The interplay of atoh1 genes in the lower rhombic lip during hindbrain morphogenesis. *PLOS ONE* **15**, e0228225 (2020).

46. R. V. Sillitoe, Y. Fu, C. Watson, in The Mouse Nervous System, C. Watson, G. Paxinos, L. Puelles, Eds. (Academic Press, San Diego, 2012), pp. 260–397.

47. C. F. Kratochwil, U. Maheshwari, F. M. Rijli, The long journey of pontine nuclei neurons: From rhombic lip to cortico-ponto-cerebellar circuitry. *Front. Neural Circuits* **11**, 33 (2017).

48. R. Machold, G. Fishell, Math1 is expressed in temporally discrete pools of cerebellar rhombic-lip neural progenitors. *Neuron* **48**, 17–24 (2005).

49. R. Chen, X. Wu, L. Jiang, Y. Zhang, Single-cell RNA-seq reveals hypothalamic cell diversity. *Cell Rep.* **18**, 3227–3241 (2017).

50. M. B. Yaylaoglu, A. Titmus, A. Visel, G. Alvarez-Bolado, C. Thaller, G. Eichele, Comprehensive

expression atlas of fibroblast growth factors and their receptors generated by a novel robotic in situ hybridization platform. *Dev. Dyn.* **234**, 371–386 (2005).

51. Y. Hao, S. Hao, E. Andersen-Nissen, W. M. Mauck III, S. Zheng, A. Butler, M. J. Lee, A. J. Wilk, C. Darby, M. Zager, P. Hoffman, M. Stoeckius, E. Papalexi, E. P. Mimitou, J. Jain, A. Srivastava, T. Stuart, L. M. Fleming, B. Yeung, A. J. Rogers, J. M. McElrath, C. A. Blish, R. Gottardo, P. Smibert, R. Satija, Integrated analysis of multimodal single-cell data. *Cell* **184**, 3573–3587.e29 (2021).

52. C. S. McGinnis, L. M. Murrow, Z. J. Gartner, DoubletFinder: Doublet detection in single-cell RNA sequencing data using artificial nearest neighbors. *Cell Syst.* **8**, 329–337.e4 (2019).

53. C. Hafemeister, R. Satija, Normalization and variance stabilization of single-cell RNA-seq data using regularized negative binomial regression. *Genome Biol.* **20**, 296 (2019).

54. G. Finak, A. McDavid, M. Yajima, J. Deng, V. Gersuk, A. K. Shalek, C. K. Slichter, H. W. Miller, M. J. McElrath, M. Prlic, P. S. Linsley, R. Gottardo, MAST: A flexible statistical framework for assessing transcriptional changes and characterizing heterogeneity in single-cell RNA sequencing data. *Genome Biol.* **16**, 278 (2015).

55. U. Raudvere, L. Kolberg, I. Kuzmin, T. Arak, P. Adler, H. Peterson, J. Vilo, G:Profiler: A web server for functional enrichment analysis and conversions of gene lists (2019 update). *Nucleic Acids Res.* **47**, W191–W198 (2019).

56. J. D. Hunter, Matplotlib: A 2D graphics environment. *Comput. Sci. Eng.* **9**, 90–95 (2007).

57. S. van der Walt, J. L. Schönberger, J. Nunez-Iglesias, F. Boulogne, J. D. Warner, N. Yager, E. Gouillart, T. Yu, scikit-image contributors, Scikit-image: Image processing in Python. *PeerJ* **2**, e453 (2014).

58. S. Bolte, F. P. Cordelières, A guided tour into subcellular colocalization analysis in light microscopy. *J. Microsc.* **224**, 213–232 (2006).

59. D. Bates, M. Mächler, B. Bolker, S. Walker, Fitting linear mixed-effects models using lme4. *J. Stat. Softw.* **67**, 1–48 (2015).

60. C. R. Harris, K. J. Millman, S. J. van der Walt, R. Gommers, P. Virtanen, D. Cournapeau, E. Wieser, J. Taylor, S. Berg, N. J. Smith, R. Kern, M. Picus, S. Hoyer, M. H. van Kerkwijk, M. Brett, A. Haldane, J. F. Del Río, M. Wiebe, P. Peterson, P. Gérard-Marchant, K. Sheppard, T. Reddy, W. Weckesser, H. Abbasi, C. Gohlke, T. E. Oliphant, Array programming with NumPy. *Nature* **585**, 357–362 (2020).

61. G. Paxinos, *Atlas of the Developing Mouse Brain at E17.5, P0 and P6* (Elsevier, Amsterdam; Boston, ed. 1st, 2007), pp. xi, 353 p.

62. M. F. Rose, J. Ren, K. A. Ahmad, H.-T. Chao, T. J. Klisch, A. Flora, J. J. Greer, H. Y. Zoghbi, Math1 is essential for the development of hindbrain neurons critical for perinatal breathing. *Neuron* **64**, 341–354 (2009).
